# The context of the ribosome binding site in mRNAs defines specificity of action of kasugamycin, an inhibitor of translation initiation

**DOI:** 10.1073/pnas.2118553119

**Published:** 2022-01-21

**Authors:** Yan Zhang, Nikolay A. Aleksashin, Dorota Klepacki, Caleb Anderson, Nora Vázquez-Laslop, Carol A. Gross, Alexander S. Mankin

**Affiliations:** ^a^Department of Microbiology and Immunology, University of California, San Francisco, CA 94158;; ^b^Department of Cell and Tissue Biology, University of California, San Francisco, CA 94158;; ^c^Center for Biomolecular Sciences, University of Illinois at Chicago, Chicago, IL 60607;; ^d^Department of Pharmaceutical Sciences, University of Illinois at Chicago, Chicago, IL 60607;; ^e^California Institute of Quantitative Biology, University of California, San Francisco, CA 94158

**Keywords:** ribosome, antibiotics, translation initiation

## Abstract

Kasugamycin (KSG) is an aminoglycoside antibiotic widely used in agriculture and exhibits considerable medical potential. Previous studies suggested that KSG interferes with translation by blocking binding of canonical messenger RNA (mRNA) and initiator transfer tRNA (tRNA) to the small ribosomal subunit, thereby preventing initiation of protein synthesis. Here, by using genome-wide approaches, we show that KSG can interfere with translation even after the formation of the 70S initiation complex on mRNA, as the extent of KSG-mediated translation inhibition correlates with increased occupancy of start codons by 70S ribosomes. Even at saturating concentrations, KSG does not completely abolish translation, allowing for continuing expression of some *Escherichia coli* proteins. Differential action of KSG significantly depends on the nature of the mRNA residue immediately preceding the start codon, with guanine in this position being the most conducive to inhibition by the drug. In addition, the activity of KSG is attenuated by translational coupling as genes whose start codons overlap with the coding regions or the stop codons of the upstream cistrons tend to be less susceptible to drug-mediated inhibition. Altogether, our findings reveal KSG as an example of a small ribosomal subunit-targeting antibiotic with a well-pronounced context specificity of action.

## Significance

Several antibiotics targeting the large ribosomal subunit interfere with translation in a context-specific manner, preventing ribosomes from polymerizing specific amino acid sequences. Here, we reveal kasugamycin as a small ribosomal subunit-targeting antibiotic whose action depends on the sequence context of the untranslated messenger RNA (mRNA) segments. We show that kasugamycin-induced ribosomal arrest at the start codons of the genes and the resulting inhibition of gene expression depend on the nature of the mRNA nucleotide immediately preceding the start codon and on the proximity of the stop codon of the upstream cistron. Our findings underlie the importance of mRNA context for the action of protein synthesis inhibitors and might help to guide the development of better antibiotics.

Translation initiation is a critical checkpoint for regulating protein synthesis. As the rate-limiting step of translation, the initiation phase is targeted by many posttranscriptional mechanisms tuning gene expression (1–3) and often serves as an important drug target (4). In bacteria, initiation of protein synthesis involves a stepwise assembly of the translation complex at the start codon of an open reading frame (ORF) (reviewed in Refs. 5 and 6). With the assistance of the initiation factors (IFs), the small (30S) ribosomal subunit recognizes the ribosome binding site (RBS) in messenger RNA (mRNA) and establishes codon-anticodon interactions between the initiator fMet-tRNA_i_ and the start codon, resulting in formation of the 30S initiation complex (30S IC). The recognition of RBS is facilitated by favorable mRNA folding and modulated by the interaction between a purine-rich Shine-Dalgarno sequence found upstream of the start codon of many genes and a complementary sequence at the 3′ end of the 16S ribosomal RNA (7). In the 30S IC, mRNA traverses the mRNA channel, a grove around the “neck” of the small ribosomal subunit that spans the A-, P-, and E- transfer tRNA (tRNA) binding sites (8–10). Association of the 30S IC with the large (50S) ribosomal subunit results in formation of the 70S initiation complex (70S IC). Departure of IFs and adjustment of fMet-tRNA_i_ in the P site converts the 70S IC into an elongation-competent ribosome ready for binding of aminoacyl-tRNA in the A site, formation of the first peptide bond, and translocation (11).

**Fig. 1.**
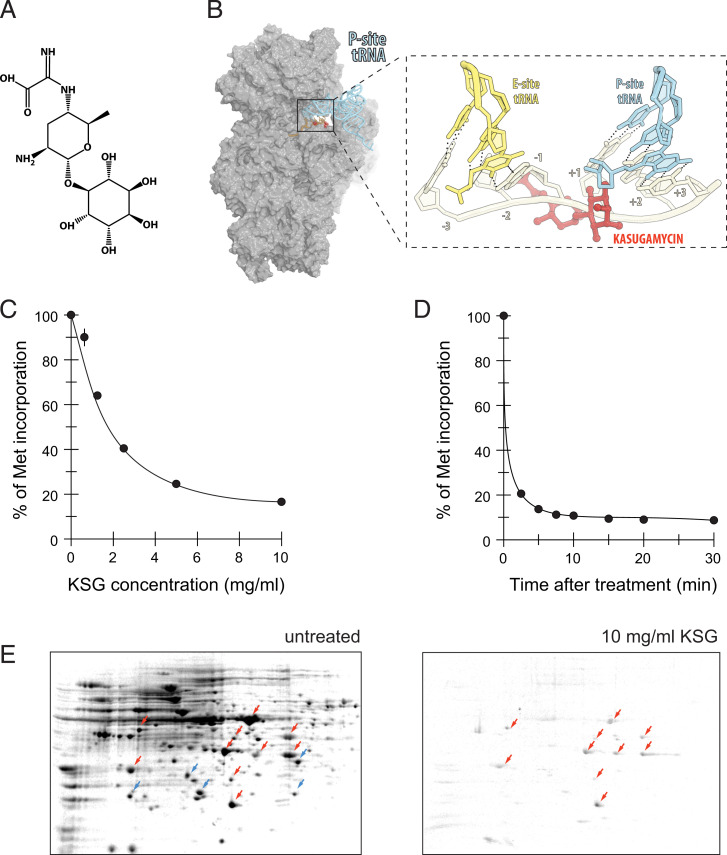
Some proteins are translated in cells exposed to high concentration of KSG. (*A*) Chemical structure of KSG. (*B*) Binding site of KSG on 30S ribosomal subunit [alignment of the structures of the *E. coli* ribosome-KSG complex (PDB 4V4H) (21) with the structure of elongating ribosome (PDB 5JTE) (58)]. The zoomed-in view shows the expected clash between the antibiotic and mRNA nucleotides preceding the start codon. (*C* and *D*) Residual protein synthesis, measured by [^35^S]-L-methionine incorporation, upon treatment of growing *E. coli* MG1655 cells with either (*C*) different concentrations of KSG for 5 min or (*D*) 10 mg/mL (100× MIC) of KSG for varying times. Translation level was normalized to that in the untreated cells. (*E*) The 2D gel electrophoretic analysis of proteins synthesized in untreated cells (“untreated”) or cells exposed for 3 min to 100× MIC of KSG. Red arrows indicate spots corresponding to the proteins whose translation continues; blue arrows indicate proteins whose translation is abolished in KSG-treated cells. By adjusting the contrast of the 2D gel image, many more protein spots can be detected in the sample prepared from KSG-treated cells (*SI Appendix,* Fig. S1).

The aminoglycoside kasugamycin (KSG), produced by *Streptomyces kasugaensis*, was one of the first translation initiation inhibitors discovered (12) (Fig. 1*A*). KSG is used as a fungicide in agriculture (13–15) and also inhibits growth of various bacteria, including important human pathogens, while exhibiting low toxicity against humans and animals (13, 16, 17). Pioneering in vitro studies of Okuyama et al. demonstrated that KSG interferes with formation of 30S IC and 70S IC on phage mRNAs (18, 19); subsequent studies confirmed this activity using a limited number of specific mRNA templates in cell-free translation systems (20, 21).

Early genetic and biochemical data revealed the small ribosomal subunit as the target of KSG action (22–24). Later on, crystallographic studies showed that KSG binds in the E site of the mRNA channel (20, 21). Structural modeling suggested that in the initiating ribosome, the antibiotic would clash with two mRNA residues in the positions −2 and −1 of the E-site codon and would also encroach upon the backbone atoms of the first residue of the P-site start codon (20, 21) (Fig. 1*B*). The predicted clash between KSG and mRNA was consistent with inhibition of initiation complex formation observed in the in vitro experiments (18–21). Even though a second antibiotic binding site was observed in the *Thermus thermophilus* 30S subunit (20), its functional relevance remains unclear. The structural data also provide some rationale for the earlier reports that suggested that translation of leaderless mRNAs could be less sensitive to inhibition by KSG (25, 26). Thus, structural and biochemical data seem to be consistent in picturing KSG as a protein synthesis inhibitor that would inhibit translation of all canonical mRNA by interfering with the formation of the 30S IC (27).

Some evidence suggested, however, that KSG effects on translation initiation might be more nuanced and possibly involve the context specificity observed for some other ribosome-targeting antibiotics (28). Even very early reports hinted that the extent of KSG inhibition was influenced by the nature of the mRNA template, as KSG was reported to differentially inhibit translation of distinct phage proteins (19). Furthermore, synthesis of *Escherichia coli* proteins was found to be abrogated by the antibiotic more readily than translation of phage polypeptides (29, 30). Prolonged treatment of *E. coli* with KSG at near minimal inhibitory concentration (MIC) of the drug was found to result in selective synthesis of some proteins possibly due to stress-induced shortening of 5′ untranslated regions (UTRs) of their mRNAs and also generation of aberrant ribosomes (31, 32). Subsequent translatome studies also suggested that expression of *E. coli* proteins was differentially affected by near-MIC concentrations of KSG, but no dependence of KSG sensitivity on the length of 5′ UTR was observed, and no specific motif that would correlate with the drug action emerged (33). On the other hand, studies using engineered translation reporters suggested a possible influence of the identity of the start codon and of the three preceding nucleotides on the extent of KSG inhibition (21). While all these observations indicated that KSG may differentially modulate expression of individual proteins, the trends underlying the specificity of KSG action and the features of cellular mRNAs that control sensitivity to the drug have remained unknown.

To unravel the principles of gene specificity of KSG action, we used genome-wide analyses to examine the effect of KSG upon cellular translation. We observed a strikingly differential effect of the antibiotic on expression of individual genes and identified the context signatures that influence the extent of ribosome inhibition by this antibiotic. The results of our studies reveal the influence of mRNA sequence upon action of the ribosome-targeting antibiotics.

## Results

### Treatment of Cells with High Concentrations of KSG Allows for Continued Translation of a Subset of Proteins.

Most previous studies of KSG action followed translation of a few specific genes, usually in cell-free translation systems, thereby precluding determining the full range of the antibiotic’s effects on endogenous mRNA translation. To overcome these limitations, we used genome-wide approaches to obtain an unbiased view of how KSG affects translation of a broad variety of individual cellular genes.

We first assessed how exposure to KSG alters overall protein synthesis. It had been observed in previous studies that synthesis of some proteins persisted in bacterial cells exposed to near-inhibitory KSG concentrations (32, 33). Therefore, we first asked whether translation could still take place in cells treated with very high concentrations of KSG. Remarkably, exposure of *E. coli* cells, strain MG1655, to even very high concentrations of KSG (10 mg/mL, ∼100-fold higher than the MIC) failed to completely abolish translation, which continued at ∼10% of that in untreated cells even after a prolonged exposure to the antibiotic (Fig. 1 *C* and *D*). This unexpectedly significant level of residual protein synthesis at high antibiotic concentrations could result from a generally inefficient inhibition of global translation or from the existence of a specific subset of polypeptides whose synthesis is resistant to the KSG treatment. To distinguish between these possibilities, we analyzed polypeptides that continued to be synthesized in the KSG-treated cells. After incubating cells for 5 min with 100× MIC of KSG, the translated proteins were pulse-labeled with [^35^S]-L-methionine and resolved by two-dimensional (2D) gel electrophoresis. The radiograms of the gels exposed for the same amount of time showed the presence of distinct radioactive spots representing a subset of polypeptides actively expressed in the KSG-treated cells (red arrows in Fig. 1*E*); a number of radioactive spots representing other proteins expressed at a lower level were also readily noticeable (*SI Appendix*, Fig. S1). Other polypeptides, actively translated in the untreated cells, were not radiolabeled in the KSG-treated cells (blue arrows in Fig. 1*E*), indicating that their synthesis was significantly inhibited by the drug. This result demonstrated that synthesis of distinct cellular proteins could continue in cells exposed to high concentrations of KSG. Compared to only a few leaderless mRNA reported to be transcribed from the *E. coli* MG1655 genome (34, 35), a much higher number of specific proteins seemed to be actively expressed in KSG-treated cells (Fig. 1*E*). Therefore, our results argue that KSG acts as a gene-specific inhibitor of translation of canonical mRNAs, curtailing synthesis of some proteins while affording active translation of others.

### Ribosome Profiling Results Challenge the Conventional Model of KSG Action.

To obtain a more detailed view of the effect of KSG upon translation of a broad array of bacterial genes, we used ribosome profiling (Ribo-seq), a technique that employs deep sequencing of the ribosome-protected mRNA fragments (ribosomes footprints [rfps]) to monitor the progression of translating ribosomes along mRNA (36, 37). In these experiments, we used the KSG-hypersusceptible Δ*gcvB* mutant of *E. coli* (38), which allowed us to reach ∼1,000× MIC when the cells were exposed to 10 mg/mL of KSG. In addition, we also collected Ribo-seq data from cells exposed to 100× MIC (1 mg/mL) of the drug. Since the Δ*gcvB* mutant is characterized by a faster uptake of the drug (38), we were able to reduce the time of antibiotic treatment at 1,000× MIC to 2.5 min to minimize the effects of secondary responses induced by persistent translation inhibition while still achieving the plateau levels of residual translation (*SI Appendix*, Fig. S2*A*).

**Fig. 2.**
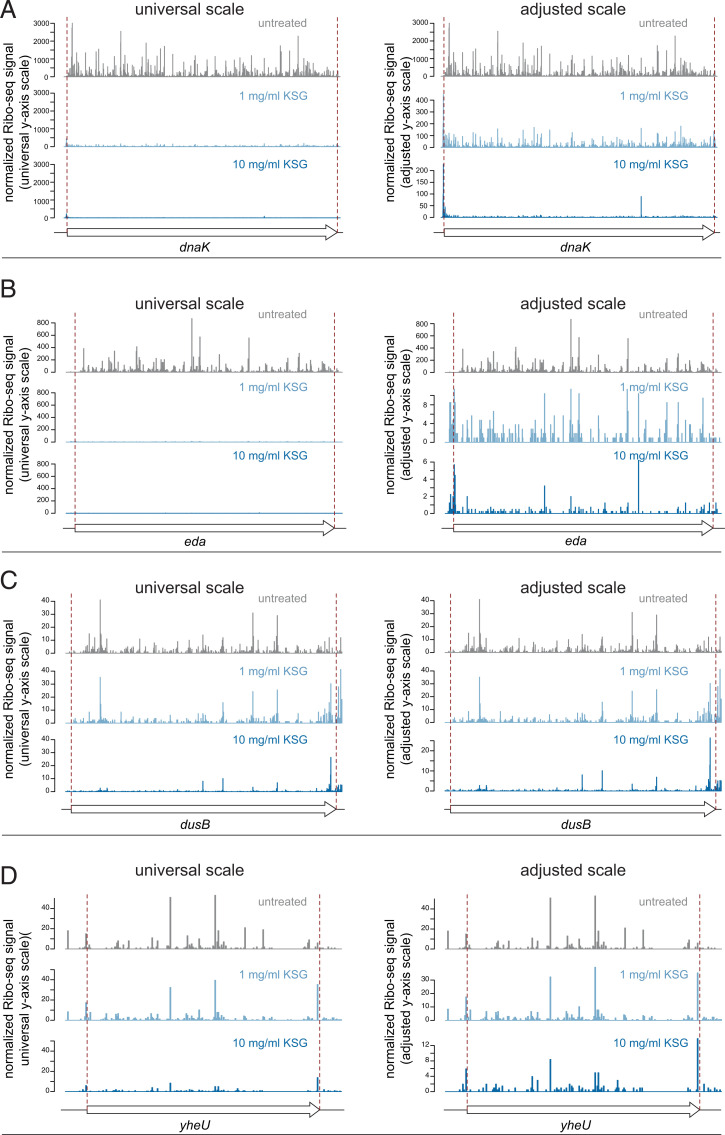
Diverse effect of KSG on translation of *E. coli* genes. Examples of genes whose translation is strongly inhibited by KSG (*A* and *B*) or only moderately affected by the drug (*C* and *D*). For each gene, the relative ribosome occupancies for the control and antibiotic-treated samples are shown using the universal *y* axis scale (*Left*) or adjusted *y* axis scales (*Right*), which reveals residual translation of the ORF. The relative ribosome occupancy (normalized Ribo-seq signal) was calculated from Ribo-seq signals normalized by the mRNA abundance (reads per kilobase per million [RPKM] calculated from RNA-seq) and adjusted by the overall level of translation inhibition (quantified by [^35^S]-L-methionine labeling). The beginning and the end of the coding sequence are indicated by red dashed lines.

The commonly accepted model of KSG action as a global inhibitor of initiation complex formation predicts that the addition of the antibiotic to growing cells would prevent new rounds of translation initiation while allowing completion of protein synthesis initiated prior to the treatment. Such runoff translation should deplete all mRNAs of ribosomes. However, the Ribo-seq analysis revealed a significantly different picture: while some ORFs indeed became almost completely depleted of rfps (Fig. 2 *A* and *B*), significant ribosome density was detected throughout the length of a number of other ORFs (Fig. 2 *C* and *D*) even in the cells treated with very high drug concentration (1,000× MIC). Consistently, following a 2.5 min of cell exposure to KSG, a reduced but nevertheless considerable level of polysomes still persisted in the cells, indicative of the active residual translation (*SI Appendix*, Fig. S2*B*). While challenging the conventional view on the mode of KSG action, these Ribo-seq results support the conclusion of our proteomics studies (Fig. 1*E*), which suggested that KSG can selectively inhibit translation of some genes, while allowing active expression of a subset of proteins.

### Start Codon Context Plays a Key Role in Gene-Specific Action of KSG.

**Fig. 3.**
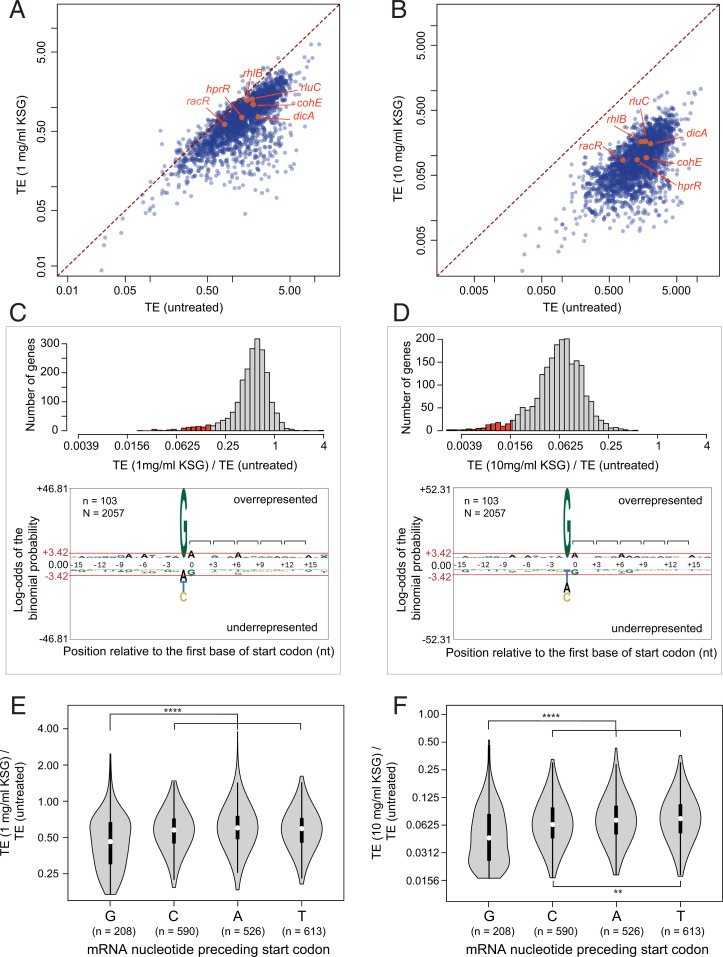
Start codon context plays a key role in gene-specific effect of KSG. (*A* and *B*) Comparison of TE of genes between untreated cells with those treated with 1 mg/mL (*A*) or 10 mg/mL (*B*) KSG for 2.5 min. The known leaderless genes, *rhlB*, *rluC*, *racR*, *hprR*, *dicA*, and *cohE* (*SI Appendix*, *Supplementary Results and Discussion*) are highlighted in orange. (*C* and *D*) (*Top*) The distribution of TE change of expressed genes (*n* = 2,057) upon treatment with 1 mg/mL (*C*) or 10 mg/mL (*D*) KSG for 2.5 min. The top 5% most inhibited genes (marked in red, *n* = 103) are characterized by the preferential occurrence of guanine immediately upstream of the start codons as revealed by pLogo analysis (59). Brackets in the pLogo plots mark the mRNA codons. (*E* and *F*) Violin plots comparing the distribution of TE changes upon 1 mg/mL (*E*) or 10 mg/mL (*F*). This analysis excludes the genes most susceptible to KSG inhibition (top 5%, highlighted in *C* and *D*). Significance values from pair-wise Mann–Whitney *U* test with Bonferroni adjustment are indicated as **P < 0.01; ****P < 0.0001.

Shortening the KSG treatment time was expected to minimize the stress-induced secondary effects observed with prolonged exposure to the antibiotic (32). However, even after the short 2.5 min of incubation with KSG, we noted changes in abundance of mRNAs transcribed from some genes (*SI Appendix*, Fig. S3 and *Supplementary Results and Discussion*). To account for these effects when assessing gene-specific action of KSG, we analyzed drug-induced changes in translation efficiency (TE), which is calculated as a ratio between the number of Ribo-seq footprints and RNA sequencing (RNA-seq) reads within protein coding sequences. TE reflects the relative number of ribosomes translating individual mRNA cistrons, and its change reveals altered translation of mRNA independent of its abundance (37). Comparison of gene-specific TE values in drug-exposed and untreated cells showed that KSG generally decreased translation of the bulk of the *E. coli* genes in the concentration-dependent manner, including that of previously reported leaderless mRNAs (Fig. 3 *A* and *B*). Translation of a majority of genes was affected at 1 mg/mL of KSG (∼100× MIC) (Fig. 3 *A* and *C*, *Top*), and the inhibition became more pronounced at 10 mg/mL (∼1,000× MIC) of the drug (Fig. 3 *B* and *D*, *Top*). However, the magnitude of the antibiotic effects varied significantly between individual genes, leading to the significant dispersion of the data points in the TE plots (Fig. 3 *A* and *B*).

We asked which features determine the sensitivity of an ORF translation to KSG inhibition. Because KSG is expected to act primarily as an initiation inhibitor, we examined the mRNA sequences in the vicinity of the start codons (positions −15 to +17 relative to the first nucleotide of the protein coding sequence). pLogo analysis of the ORFs most significantly affected by the drug (top 5%, 103 out of 2,057 analyzed genes) (Fig. 3 *C* and *D*) showed the striking prevalence of a guanine residue at position (−1), immediately upstream of the start codon (Fig. 3 *C* and *D*). To determine whether KSG-mediated inhibition of translation correlates with the presence of G(−1) throughout the entire spectrum of expressed genes, we analyzed the change in TE elicited by KSG across the remaining translated ORFs (*n* = 1,937). Consistent with the preferential presence of G(−1) in the most affected genes (Fig. 3 *C* and *D*), the differential TE analysis of the remaining 95% of the translatome demonstrated that KSG more efficiently inhibits the translation of the genes whose start codons are immediately preceded by a G compared to those where A, C, or U are found at position −1 (Fig. 3 *E* and *F*). Additionally, a C in the −1 position is marginally more sensitive to KSG than A or U.

**Fig. 4.**
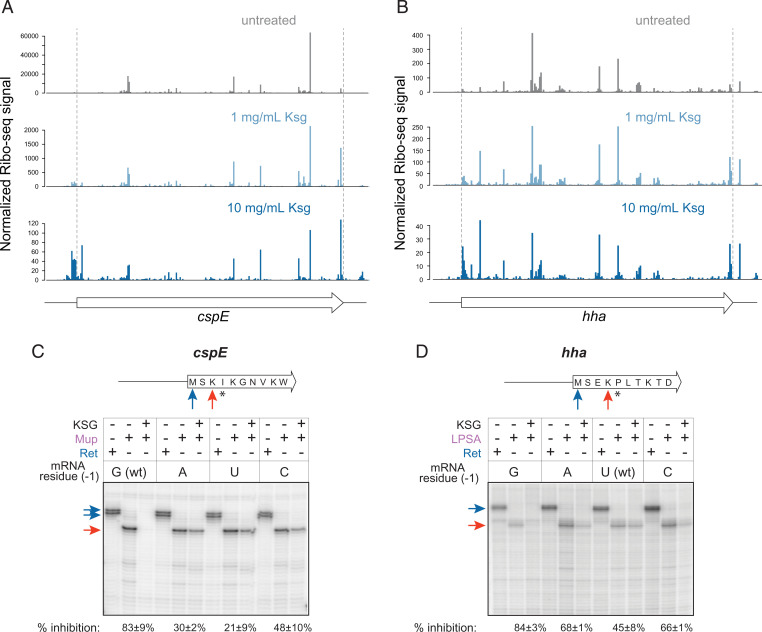
The identity of the nucleotide preceding the start codon affects KSG action in vitro. (*A* and *B*) Relative ribosome occupancy in vivo throughout the length of the *cspE* (*A*) and *hha* (*B*) ORFs in untreated cells and those treated with 1 mg/mL or 10 mg/mL of KSG for 2.5 min. The relative ribosome occupancies (normalized Ribo-seq signal) are shown using adjusted *y* axis scales. The locations of the first base of start codon and the last base of stop codon are highlighted by the dashed red lines. (*C* and *D*) Toeprinting analysis of inhibition of translation by KSG. (*Top*) Schematics of mRNA templates used for in vitro translation: both the *hha* and *cspE* RNA transcripts contained 18 codons of the respective genes preceded by 22 nt (*hha*) or 60 nt (*cspE*) of the native 5′ UTR (see *SI Appendix*, Table S3 for details). Blue arrows indicate the start codons positioned in the P site of the initiating ribosome; asterisks indicate the “hungry” codons generated by using the inhibitors of the respective aminoacyl-tRNA synthetases; orange arrows indicate the codon in the P site of the ribosome trapped immediately upstream of the hungry codon. (*Bottom*) Toeprinting gels reflecting ribosome arrest during translation of the *cspE* (*C*) and *hha* (*D*) genes with mutations of the mRNA nucleotide preceding the start codon [“mRNA residue (−1)”]. Ile-RS inhibitor mupirocin (Mup) or Pro-RS inhibitor LPSA were present in the toeprinting reactions with *cspE* and *hha* templates, respectively. Inhibition of translation by KSG allows fewer ribosomes to reach the trap codon resulting in the decreased intensity of the trap codon band (orange arrow). The control antibiotic retapamulin (Ret) arrests the ribosome at the start codon (blue arrow) (52).

To directly examine the influence of the mRNA residue preceding the start codon on KSG action, we selected two representative genes that showed significantly differential susceptibility to KSG in the cells exposed to the drug: *cspE*, which carries G(−1) and whose in vivo translation was nearly abolished at 10 mg/mL of KSG (Fig. 4*A*), and *hha* with U(−1), whose translation in the presence of the drug continued at a considerable level compared to the untreated control (Fig. 4*B*). We then used in vitro toeprinting analysis, which allows monitoring progression of ribosomes along mRNAs (39, 40), to test whether changes of a single mRNA residue preceding the start codon would influence the response to KSG in a cell-free translation system. To account for the ribosomes that were able to successfully initiate translation, we trapped elongating ribosomes at a specific “hungry” codon of the mRNA generated by depleting the translation reactions of specific aminoacyl-tRNAs (41) (Fig. 4 *C* and *D*). In vitro translation of wt *cspE* with the native G(−1) was readily inhibited by KSG, as very few ribosomes reached the Ile4 trap codon when KSG was present in the reaction (Fig. 4*C*). Mutating G(−1) of *cspE* to A, U, or C diminished the effect of the drug as notably more ribosomes translated up to the trap codon (Fig. 4*C*). Conversely, translation of the *hha* ORF with the wt U(−1) or the A(−1) or C(−1) mutations was only modestly affected by KSG, while mutating U(−1) to G significantly sensitized *hha* translation to the antibiotic inhibition, as judged from the disappearance of the toeprint bands at the “hungry” Pro5 codon (Fig. 4*D*). Consistent with the results of the in vivo Ribo-seq experiment (Fig. 3 *E* and *F*), a C at the −1 position results in slightly more severe KSG inhibition of *cspE* translation than A or U.

Taken together, our in vivo (Ribo-seq) and in vitro (toeprinting) data strongly argue that the identity of the mRNA nucleotide immediately upstream of the start codon greatly influences the inhibitory action of KSG; G(−1) emerged as a key determinant of susceptibility to KSG.

### KSG Differentially Affects Initiation of Translation of Different Genes.

**Fig. 5.**
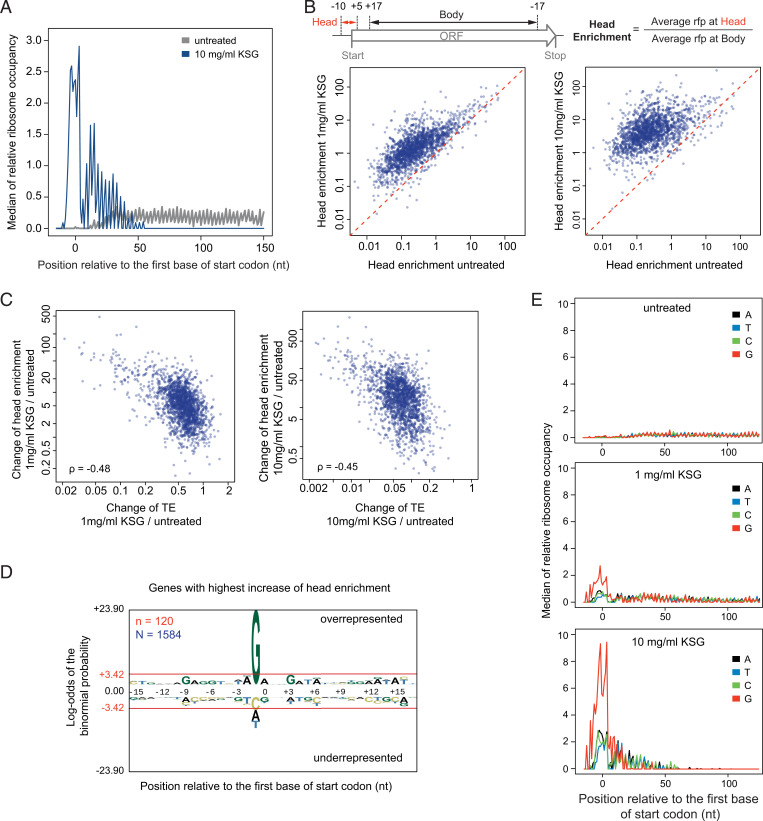
KSG treatment leads to ribosome enrichment in vicinity of the start codon. (*A*) Metagene analysis (see *Materials and Methods* for details) of the median rfp density in the vicinity of start codons in untreated cells and cells treated with 10 mg/mL of KSG. (*B*) (*Top*) Schematic for calculating the head enrichment; (*Bottom*) comparison of the change in head enrichment of different genes upon treatment of 1 mg/mL KSG (*Left*) or 10 mg/mL KSG (*Right*) versus untreated control. (*C*) Comparisons of changes of TE versus changes of head enrichment upon treatment with 1 mg/mL KSG (*Left*) or 10 mg/mL KSG (*Right*). ρ: Spearman’s rank correlation coefficient. (*D*) Genes with the highest head enrichment are characterized by the preferential presence of G preceding the start codon. Sequence signatures are generated by pLogo analysis of the top 10% genes shared between the two KSG-treated samples (*n* = 120) out of 1,584 analyzed. Bases above or below the red line are significantly overrepresented or underrepresented, respectively. (*E*) Metagene analysis of the median rfp density in the vicinity of start codons in untreated and KSG-treated cells plotted in different colors for mRNAs with different residues preceding the start codon.

The conventional view of the mechanism of KSG action presumes that the drug inhibits binding of canonical (leadered) mRNAs to the small ribosomal subunit, thus interfering with formation of the 30S IC and, consequently, preventing assembly of the 70S IC at the start codon. However, in KSG-treated cells, we observed a dramatic increase of the relative start codon occupancy by 70S ribosomes across the genome (Fig. 5*A*). The high rfp density peak in the “head region” of the ORF, which encompasses the start codon, is followed by a 2 to 3 codon-wide valley, characterized by a scarce ribosomal occupancy, and a second enrichment peak at several of the following codons of the “neck region” (Fig. 5*A*).

To quantify the gene-specific change in ribosome occupancy of the start codons, we implemented the “head enrichment” metric, which measures the relative ribosome occupancy at the head region compared to that across the body of the ORF (Fig. 5*B*). KSG treatment leads to a general increase of head enrichment across the genome, with higher KSG concentration resulting in stronger increase (Fig. 5*B*). However, the magnitude of this effect differs between genes (Fig. 5*B*). Remarkably, the KSG-induced changes in the head enrichment negatively correlate with drug-induced changes in TE (Spearman’s correlation coefficient ρ = −0.48 or −0.45, with 1 mg/mL or 10 mg/mL KSG, respectively) (Fig. 5*C*), indicating that genes whose translation is more susceptible to KSG inhibition tend to have higher start codon occupancy by ribosomes in KSG-treated cells. Consistently, genes with the highest increase of head enrichment in KSG-treated cells showed a strong prevalence for G(−1) (Fig. 5 *D* and *E*), which, as we showed earlier, is the signature of the genes more susceptible to inhibition by the drug (Fig. 3 *C* and *D*). This observation suggests that, in contrast to the conventional view, KSG inhibits translation of many genes not only before but after the assembly of 70S IC, retaining the ribosome at the start codon and preventing it from progressing to the elongation stage of protein synthesis.

### Translation Coupling May Attenuate KSG Action.

**Fig. 6.**
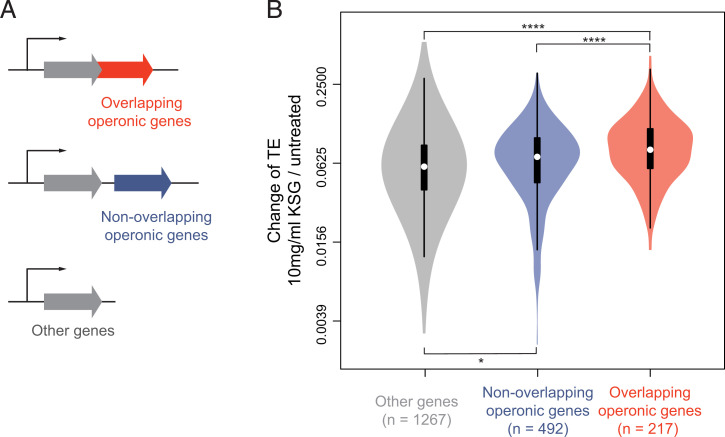
Translation coupling attenuates action of KSG on translation initiation. (*A*) *E. coli* endogenous genes were divided into three categories: overlapping operonic genes (red) where the start codon of the downstream gene overlaps with the coding sequence or the stop codon of the upstream gene in the same operon; nonoverlapping operonic genes (blue); other genes (encoded in single-gene transcription units or the first genes in polycistronic operons). See *SI Appendix*, *Supplementary Results and Discussion* for details. (*B*) Violin plots comparing changes of TE in different gene categories in cells exposed to 10 mg/mL of KSG. Significance values from pair-wise *t* test comparison with Bonferroni adjustment are indicated as ****P < 0.0001; *P < 0.05.

Translational coupling, which is defined as the interdependence of translation of adjacent cistrons on the same polycistronic mRNA, may be mediated by direct recruitment of 30S subunit or 70S ribosomes from the upstream genes to the start codon of the downstream genes (1, 42, 43). We reasoned that action of KSG upon the reinitiating ribosome might differ from its action upon the ribosome progressing through the conventional initiation pathway. To examine the genome-wide effect of translation coupling on KSG action, we compared KSG-induced TE changes in genes whose translation coupling is expected to be most pronounced (those whose start codon overlaps or even localizes upstream of a stop codon of the preceding cistron in the same operon) with those from nonoverlapping genes (Fig. 6*A*). The results show that translation of genes whose start codons overlap with the upstream cistron was less affected by KSG in comparison with the translationally uncoupled genes (Fig. 6*B*), suggesting that translational coupling could counteract the action of KSG and may account, in part, for the gene-specific action of this antibiotic.

## Discussion

Genome-wide approaches enabled us to gain important insights into the mechanism of action of KSG in the living cell. Our main findings can be summarized as follows: 1) inhibition of translation by KSG is generally associated with increased relative ribosome occupancy near the start codons of the ORFs; 2) KSG inhibits protein synthesis in a context-dependent manner, differentially affecting translation of diverse genes; 3) the nature of the mRNA residue immediately preceding the start codon significantly influences KSG action, with G being the most conducive to inhibition of translation, followed distantly by C and then by U and A; 4) translational coupling, diminishes the inhibitory effect of KSG, likely because of the difference between the de novo initiation and reinitiation pathways.

Previously proposed models of KSG action presumed that the drug prevents association of mRNA with the small ribosomal subunit, thereby inhibiting translation primarily at the stage preceding the formation of the 70S IC (18, 20, 21). While interference with the formation of the 30S IC does likely contribute to the inhibitory activity of KSG, leading to the overall decrease in TE across the genome, our data reveal an important aspect of the antibiotic action taking place after association of the small and large ribosomal subunits into 70S complexes. The observed accumulation of 70S ribosomes near the start codons of the ORFs in KSG-treated cells, which correlates with reduced TE, strongly argues that inhibition of translation by KSG involves stalling of the 70S ribosomes at the start codons, preventing them from engaging in active translation. We envision two possible scenarios that could account for this aspect of KSG action. One possibility is that the drug prevents maturation of the 70S IC and its conversion into the elongation-competent ribosome. The 70S ICs maturation is a highly dynamic process that leads to dissociation of the IFs and full accommodation of fMet-tRNA in the P site and is associated with intra- and intersubunit movements (10, 11). Conceivably, KSG could inhibit one or several of the structural transitions within the 70S IC, thereby stalling its maturation. An alternative possibility is that the drug interferes with the first elongation cycle, possibly preventing the translocation, which requires mRNA movement through the KSG-obstructed mRNA channel in the 30S subunit and repositioning of the tRNA_i_^Met^ into the E site. The lack of E-site tRNA in the initiating ribosome could explain why the antibiotic exerts its inhibitory action preferentially at the start codons. The increase in the start codon occupancy observed in our Ribo-seq experiments is compatible with either scenario because this technique does not distinguish between the rfps originating from 70S IC or elongation-competent 70S ribosomes. Interestingly, toeprinting did not show ribosome stalling at the start codons (Fig. 4 *C* and *D* and *SI Appendix*, Fig. S4*B*). This discrepancy might stem from significant differences in the kinetics of the various initiation steps in vitro versus in vivo (e.g., 30S IC and 70S IC formation/maturation or transition to elongation) or from the absence in the reconstituted cell-free translation system of cellular factors required for KSG-mediated ribosome arrest.

It is likely that in the living cell, some of the ribosomes stalled by KSG at the start codons of the ORFs never progress to translate the encoded protein and eventually dissociate from mRNA. However, a significant fraction of ribosomes can occasionally escape from the trap and engage in productive translation even at a very high concentration of KSG (1,000× MIC). The peculiar distribution of the rfp density within the first several codons of the genes revealed by the metagene analysis (Fig. 5*A*) suggests that the drug possibly remains bound during several initial rounds of elongation but then eventually gets displaced, either due to competition with the E-site tRNA or being brushed away by mRNA advancing through the mRNA channel.

One of our key findings is that KSG action is context specific. The central feature that defines KSG selectivity is the nature of the nucleotide preceding the start codon of the ORF. KSG more readily decreases TE of the genes whose start codon is preceded by a G compared to those with other residues in the equivalent position. Generally speaking, the G(−1) trend may operate at the level of formation of the 30S IC or at the later steps preceding translation elongation. While our data provide little insights into the action of the drug prior to 70S IC formation, we observed a more pronounced accumulation of 70S ribosomes at the start codons of the genes with a G(−1) (Fig. 5 *D* and *E*). Therefore, it is clear that the G(−1) facilitates the drug action upon the 70S ribosomes possibly by stimulating antibiotic binding. However, lacking the structure of the ribosome-mRNA-KSG complex, it is hard to predict the exact nature of the interactions of the antibiotic molecule with the ribosome-bound mRNA. The alignment of the atomic coordinates of initiating or elongating ribosomes with the available structures of the ribosome/KSG complex shows a clash between the antibiotic molecule and mRNA (Fig. 1*B* and *SI Appendix*, Fig. S5). Clearly, rearrangements of the mRNA trajectory or repositioning of the drug would be required to simultaneously accommodate mRNA and KSG within the mRNA channel. KSG bound in the putative second site, as was observed in the *T. thermophilus* 30S subunit (20), would also clash with mRNA. However, the existence of such binding site in the 70S ribosome and, more importantly, its functional significance remain dubious. Irrespective of the exact nature of interactions between the drug, the ribosome, and mRNA, KSG expands the growing list of antibiotics that inhibit translation in a context-specific manner (28). An important difference is that selectivity of KSG action is defined not by the nature of the nascent peptide, as was the case with previously studied context-specific inhibitors (44–46), but by the sequence of the UTR of mRNA. Similar to other context-specific antibiotics, additional factors (e.g., extended sequence context, mRNA secondary structure, kinetics of translation initiation, etc.) likely also modulate drug action. Therefore, the prevalence of G(−1) in the genes more susceptible to KSG inhibition represents a trend rather than a rule.

An additional layer of KSG selectivity stems from translational coupling. Expanding the previous general observations that translation of downstream genes in polycistronic operons tend to be less affected by KSG (33), our Ribo-seq data show that it is specifically translation of the ORFs whose start codons overlap with or precede the stop codon of the upstream cistron are less susceptible to inhibition by the drug. The mechanisms of translational coupling are unclear and may either involve canonical initiation promoted by mRNA unwinding or, alternatively, reinitiation by the 30S subunits or even by 70S ribosomes that had completed translation of the upstream ORF (43, 47–49). The differential response of translationally coupled upstream and downstream ORFs to KSG is more compatible with the reinitiation scenario, which likely involves different conformational states of the reinitiating 70S ribosome or the 30S subunit in comparison with the canonical initiation pathway.

Some of the earlier studies pointed to a contrasting effect of KSG on translation of the leadered (canonical) and phage-encoded leaderless mRNAs, with the latter ones being less affected by the antibiotic (21, 25, 26). It was proposed that translation initiation of the leaderless transcripts is less sensitive to KSG inhibition because it may rely on a direct interaction of the tight-coupled 70S ribosome with the start codon at the mRNA 5′ terminus, whereas initiation at the leadered mRNAs is more sensitive to the drug as it proceeds through the formation of the 30S IC (31, 50, 51). In our genome-wide analysis, the leaderless mRNAs that are known to be transcribed from the *E. coli* genome (34, 35) did not stand out from mRNAs with the canonical 5′ UTRs, and their translation was as sensitive to KSG inhibition as that of the bulk of the *E. coli* ORFs (Fig. 3 *A* and *B*). This result corroborates the earlier finding that treatment of *E. coli* with KSG did not lead to preferential exclusion of the leadered mRNAs from polysomes in comparison with the leaderless transcripts (33) as well as the observation that reporters encoded in the leaderless and leadered mRNAs showed comparable susceptibility to the drug (17). It is possible that the 70S ribosome-based initiation mode plays only a limited role in translation of the endogenous *E. coli* leaderless transcripts or that additional cellular factors sensitize 70S-based translation initiation to KSG. We did not find support for the proposal that preferential translation of specific proteins in KSG-treated cells is mediated by shortening of 5′ UTRs of the corresponding mRNAs (32): while we observed robust selective residual translation of some proteins, our RNA-seq data did not reveal any 5′ UTR shortening upon brief exposure of *E. coli* to high concentrations of the inhibitor. In fact, we did not detect any dependence of KSG-mediated inhibition of translation on the length of 5′ UTR genome-wide (*SI Appendix*, Fig. S6). Our data strongly argue that gene selectivity of KSG action is manifested with the canonical mRNAs.

**Fig. 7.**
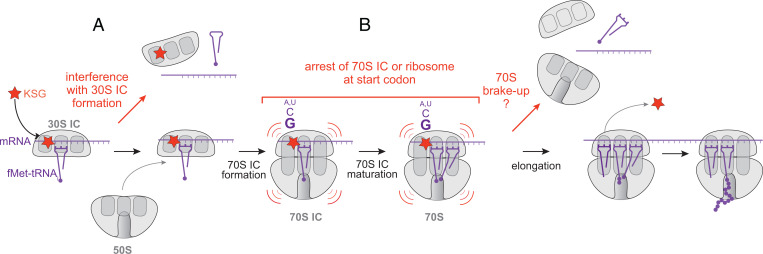
The general model of KSG action. (*A*) Binding of KSG to the small ribosomal subunit partially interferes with formation of the 30S IC; (*B*) KSG blocks either maturation of 70S IC or the first rounds of elongation, thereby arresting the 70S translation complex at the start codon. The nature of the mRNA residue preceding the start codon affects the severity of translation arrest, with G being most conducive to antibiotic action. KSG-arrested ribosomes may dissociate from mRNA or, occasionally, proceed to synthesize the encoded protein. In the latter case, the KSG molecule is likely displaced at the early rounds of translation.

Our findings allow us to propose the general model of KSG action which synthesizes our results and the previous findings (Fig. 7). According to the proposed model, KSG can interfere with initiation of translation at two different stages: A) binding of the antibiotic to the 30S subunit likely obstructs the formation of the 30S IC occasionally aborting translation at this stage; the action of antibiotic upon 30S IC may or may not be context-dependent; and B) a significant fraction of KSG-bound 30S IC can associate with the 50S subunit and proceed to form 70S IC. KSG arrests 70S complex at the start codon either by interfering with the maturation of 70S IC or by preventing the first round of elongation. The inhibitory action of the drug upon the 70S complex is stimulated by a guanine residue preceding the start codon, with cytidine being the remote second best. Some of the KSG-arrested 70S ribosomes likely eventually dissociate from mRNA, but others proceed to translate the ORF. The KSG molecule dissociates from the elongating ribosome at the early rounds of elongation, being displaced by the E-site tRNAs or brushed away by the mRNA progression.

## Materials and Methods

### Residual Translation Measured by [^35^S]-L-Methionine Incorporation.

The inhibition of protein synthesis by KSG was analyzed by metabolic labeling as described previously (52), with the minor modifications described in *SI Appendix*, *Supplementary Materials and Methods*.

### 2D Gel Electrophoretic Analysis of the Radiolabeled Proteins.

Total *E. coli* protein was isolated from the 50 mL exponentially growing *E. coli* culture (strain MG1655) as described previously (53). The experimental details can be found in *SI Appendix*, *Supplementary Materials and Methods*. The radiolabeled proteins were isolated from the 60 μL exponential cultures exposed for 3 min to 10 mg/mL of KSG, then incubated with 10 µCi of L-[^35^S]-methionine (specific activity 1,175 Ci/mmol, MP Biomedicals) and quenched after 3 min with an excess of unlabeled L-methionine.

The isolated proteins were resolved following the 2-D Electrophoresis Workflow manual (fourth edition) (BioRad). See *SI Appendix*, *Supplementary Materials and Methods* for experimental details.

### Ribo-seq and mRNA-seq.

Ribo-seq was performed following the procedure described in Ref. 37. The experimental details can be found in *SI Appendix*, *Supplementary Materials and Methods*. Total RNA for the RNA-seq analysis was phenol extracted from the same lysate that was used for Ribo-seq. Short RNA and ribosomal RNA were removed from the total RNA with MEGAclear transcription clean-up kit (Invitrogen, AM1908) and MICROBExpress bacterial mRNA enrichment kit (Ambion, AM1905), respectively. RNA was fragmented using RNA Fragmentation Reagents (Ambion, AM8740) by incubating at 95 °C for 1 min 45 s. The RNA fragments were separated in Novex 15% TBE-Urea gel (Invitrogen, EC6885BOX). The fragments in the 25 to 45 nt range were excised and converted to the sequencing library using the same strategy as for Ribo-seq (*SI Appendix*, *Supplementary Materials and Methods*).

### Ribo-seq and RNA-seq Data Analysis.

Raw reads were filtered for quality and the linker sequence was removed using FASTX-Toolkit (http://hannonlab.cshl.edu/fastx_toolkit/). The sequences were then mapped to the reference *E. coli* MG1655 genome (NC_000913.2.fna) obtained from the National Center for Biotechnology Information (NCBI) Reference Sequence Bank using Bowtie version 1.2.3 (http://bowtie-bio.sourceforge.net/index.shtml), allowing for no more than two mismatches. Reads mapped to more than one locus were discarded. Ribosome density was assigned to the 3′ end of the reads and then adjusted by a shift of −12 nt so it reflects the location of the first base of A-site codon. RNA-seq reads were similarly mapped to *E. coli* MG1655 genome. Uniquely mapped reads were equally assigned to all the bases that each read covers. For example, for a RNA-seq read that is *N*-nt long, all the bases covered by the read were given a score of 1/*N*.

The other details of bioinformatics analysis can be found in *SI Appendix*, *Supplementary Materials and Methods*.

### Toeprinting Analysis.

The DNA templates for toeprinting (*SI Appendix*, Table S3) were generated by PCR as described in *SI Appendix*, *Supplementary Materials and Methods* using respective primers listed in *SI Appendix*, Table S4. Toeprinting analysis was carried out as described previously (41, 54). The final concentration of KSG in the toeprinting reactions was 50 µM (Fig. 4 *C* and *D*) or 1 mM (*SI Appendix*, Fig. S4*B*). When needed, the Pro-RS inhibitor 5′-O-[*N*-(L-prolyl)-sulfamoyl] adenosine (55) or the Ile-RS inhibitor mupirocin (56) were added to the reactions to trap the ribosome at a specific “hungry” mRNA codon (57). These inhibitors as well as the control antibiotic retapamulin were present in the reactions at 50 µM.

### Figure Preparation.

Figures showing ribosome structures were prepared in PyMOL (Molecular Graphics System, Version 2.0 Schrödinger, LLC.)

## Supplementary Material

Supplementary File

## Data Availability

The Ribo-seq and RNA-seq data have been deposited in the NCBI Gene Expression Omnibus database under accession code GSE185757 (https://www.ncbi.nlm.nih.gov/geo/query/acc.cgi?acc=GSE185757).
