# Supplementary material for: The context of the ribosome binding site in mRNAs defines specificity of action of kasugamycin, an inhibitor of translation initiation
Source: Proc Natl Acad Sci U S A. 2022 Jan 21;119(4):e2118553119. doi: 10.1073/pnas.2118553119 (PMC8794815; doi:10.1073/pnas.2118553119)
Supplement: Supplementary File [file pnas.2118553119.sapp.pdf]

SUPPLEMENTARY INFORMATION

Supplementary Results and Discussion

Rapid transcriptional response to the KSG presence

Even with a very brief treatment of cells with KSG, we still observed certain changes in mRNA abundance indicating that *E. coli* cells rapidly respond to antibiotic exposure by remodeling its transcriptome (Fig. S3, Tables S1 and S2). Abundance of mRNAs transcribed from the genes involved in response to oxidative stresses (e.g. *soxS*, *dps*, *grxA*, *trxC*) (S6) was notably increased, in agreement with previous studies showing that multiple antibiotics induce redox-related physiological alterations (1). The high concentration of KSG also strongly induced transcription of the *csp* family proteins that are responsive to cold shock (e.g., *cspB* and *cspG*) (Fig. S3) but not those constitutively expressed at the optimal temperature (e.g. *cspC* and *cspE*). Conversely, methionine biosynthesis genes were downregulated at 10 mg/mL KSG (Fig. S3). These results suggest that KSG treatment leads to a rapid cellular response potentially resulting from the inhibition of translation initiation, the availability of a free ribosome pool, the shift of charged tRNA pool, or the genome-wide change of RNA stability due to the run-off of elongating ribosomes. Nevertheless, when we inactivated the top up-regulated gene, *soxS*, the MIC of KSG did not change indicating that the general stress response, adapted for a broad range of environmental conditions, does not facilitate notably cell survival in the presence of this antibiotic.

Supplementary Materials and Methods

Residual translation measured by [^{35}S]-L methionine incorporation

The inhibition of protein synthesis by kasugamycin was analyzed by metabolic labeling as described previously (2) with the minor modifications. Cells were grown overnight at 37°C in MOPS medium lacking methionine (MOPSΔMet) (Teknova). Cells were diluted 1:100 into fresh medium and grown at 37°C until the culture density reached A_{600}~0.4. Subsequent operations were performed using Eppendorf incubator-shaker set at 37°C. The aliquots of cell culture (400 µL) were transferred to Eppendorf tubes containing appropriately diluted KSG in MOPSΔMet medium to obtain the required final concentrations of the drug in the total volume of 500 µL. After incubating cells with antibiotic for 5 min with shaking, 30 ul of cells was transferred to a tube containing 5 µL of MOPSΔMet medium supplemented with 0.3 µCi of L-[^{35}S]-methionine (specific activity 1,175 Ci/mmol, MP Biomedicals). After 3 min incubation, unlabeled L-methionine (Sigma) was added to the final concentration of 1 mM. After 3 min incubation, the mixture was transferred onto 35 mm 3MM paper discs (Whatman) pre-wetted with 5% TCA. After collecting all the experimental points, the discs were then placed in a beaker with 500 mL 5% TCA and boiled for 5 min. TCA was discarded and this step was repeated one more time. Discs were rinsed in acetone, air-dried and placed in scintillation vials. After addition of 5 mL of scintillation cocktail (Perkin Elmer, Ultima Gold) the amount of retained radioactivity was measured in scintillation counter (Beckman). The data obtained from KSG-treated cells were normalized to the no-drug control.

The time course of inhibition of protein synthesis by kasugamycin was monitored following essentially the same procedure except that antibiotic was added to a tube with the cells and 30 µL aliquots were withdrawn after specified time, incubated for 3 min with 5 µL of L-[^{35}S]-methionine, followed by 3 min incubation with cold L-methionine. The rest of the steps were as described above.

2D-gel electrophoretic analysis of the radiolabeled proteins

<u>Isolation of total *E. coli* protein</u>

E. coli cells (strain MG1655) were grown overnight at 37°C in LB medium. Cultures were diluted 1:200 into 50 ml of fresh medium and grown at 37°C until the culture density reached A_{600}~0.5. The cells were pelleted by centrifugation at 5000 g for 5 min at 4°C. Cells were washed twice with washing buffer (10 mM Tris-HCl, pH 7.0, 250 mM sucrose), resuspended in 600 µL of lysis buffer (100 mM Tris-HCl, pH 9.5, 1% SDS) and lysed by incubating at 95°C for 5 min. Cell lysates were then sequentially supplemented with 2.4 mL of methanol, 600 µL of chloroform and 1.8 mL of H_2O and vortexed. The samples were centrifuged at 14,000 g for 5 min at room temperature. The upper (aqueous) fraction was removed and proteins were precipitated by addition of 2.4 mL of methanol to the remaining solution. Protein pellets were collected by centrifugation at 14000 g for 5 min at room temperature. The pellets were dissolved in 200 µL of freshly prepared urea/thiourea buffer (7 M urea, 2 M thiourea, 40 mM DTT). Total proteins were re-precipitated by adding 1.3 mL of cold (-20°C) acetone. After 1 h incubation at -20°C, samples were centrifuged at 14,000 g for 10 min at 4°C. The pellets were washed twice with 75% cold (-20°C) acetone. The pellets were then air dried and resuspend in 100 µL of 2D-rehydration/sample buffer (Bio-Rad) to the final concentration of 10 mg/ml. The protein concentration was estimated by Bradford assay (3).

Metabolic labeling of proteins for 2D-Gel Electrophoresis Analysis

E. coli cells (strain MG1655) were grown overnight at 37°C in MOPS medium lacking methionine (MOPSΔMet) (Teknova). Cells were diluted 1:200 into fresh medium and grown at 37°C until the culture density reached A_{600}~0.3. Subsequent operations were performed at 37°C. The aliquots of cell culture (60 µL) were transferred to Eppendorf tubes containing 13 mg/ml of KSG in the total volume of 200 µL of MOPSΔMet medium (the final KSG cocnemntration is 10 mg/ml). In the control samples, KSG was omitted. After incubating cells with antibiotic for 3 min, 10 µCi of L-[^{35}S]-methionine (specific activity 1,175 Ci/mmol, MP Biomedicals) was added. After 3 min incubation, non-radioactive L-methionine (Sigma) was added to the final concentration of 1 mM in order to quench incorporation of radioactivity into protein. The cells were pelleted by centrifugation at 5,000 g for 5 min at 4°C. The pellet was resuspended in 15 µL of lysis buffer (1% SDS, 100 mM Tris-HCl, pH 9.5) and cells were lysed by boiling at 95°C for 5 min.

2D separation of proteins

Separation of proteins in the first dimension was achieved by isoelectrofocusing. 0.5 mg of total *E. coli* proteins were combined with ~ 0.5 μCi of L-[^{35}S]-methionine-labeled proteins in 315 μL of 2D-rehydration/sample buffer (Bio-Rad). The 18 cm ReadyStri IPG pH 4-7 IEF strip (Bio-Rad) was placed on top of the 2D-rehydration solution in rehydration tray. After addition of 7 ml of mineral oil on top of the strip, the tray was incubated at room temperature for 18 h. The strip was placed in isoelectrofocusing tray (BioRad) and covered with mineral oil. The isoelectric focusing was performed in 4 steps according to the manufacture instructions (step 1 – 250 V, rapid ramp for 30 min, step 2 – 10,000 V gradual ramp for 2 h, step 3- 10,000 V rapid ramp 43,000 V/h, step 4 – 10,000 V infinitely long). The mineral oil was blotted and proteins on the strip were reduced by incubation for 20 min in 5 mL of reduction solution (50 mM Tris-HCl, pH 6.8, 6 M urea, 30% Glycerol, 0.01% SDS, 65 mM DTT). The reduction solution was removed, and strip was incubated for 20 min in 5 mL of alkylation solution (50 mM Tris-HCl, pH 6.8, 6 M urea, 30% glycerol, 0.01% SDS, 245 mM iodoacetamide, 0.0002% bromophenol blue). After removal of the alkylation solution, the strip was equilibrated for 1 h with cathode buffer (100 mM Tris-HCl, pH 8.25, 100 mM Tricine, 0.3% SDS).

Separation of proteins in the second dimension was performed by electrophoresis in 20x20 cm 12% PAAG SDS gel following the published protocol (4) except that the cathode buffer was supplemented with 0.05% thioglycolic acid (Sigma). The electrophoresis was run at 200 V until the bromophenol blue dye reached the bottom of the gel. Gels were fixed with 5% perchloric acid, stained with Coomassie blue, dried, and exposed overnight to a phosphorimager screen. Radioactivity was visualized in a Typhoon Trio scanner (GE Healthcare).

Ribo-seq

Cell growth and RNA preparation

Ribo-seq experiments were carried out following the protocol for bacterial ribosome profiling described in (5). *E. coli* MG1655 Δ*gcvB* cells were grown overnight at 37˚C in MOPS rich defined medium lacking methionine (Teknova). The cultures were diluted to an OD_{600} = 0.005 in fresh medium and grown until OD_{600} reached 0.35. For KSG-treated samples, KSG powder was

dissolved in medium and added to log-phase culture to final concentration of 1 mg/mL or 10 mg/mL. Cultures were kept shaking at 37˚C for 2.5 min. 200 mL of cell culture was rapidly filtered by passing through a Whatman cellulose nitrate membrane filter with 220 nm pore size (GE Healthcare, 7182-009) and the cell pellet was flash-frozen in liquid nitrogen. Cells were pulverized in 10mL canisters pre-chilled in liquid nitrogen with 600 µL of frozen lysis buffer (10 mM $MgCl_2$, 100mM NH_4Cl, 20mM Tris-HCl pH 8.0, 0.1% NP-40, 0.4% Triton X-100, 100 U/mL DNase I (Roche, 4716728001), 1mM chloramphenicol) using QIAGEN TissueLyser II (5 cycles of 3 min at 15 Hz). Pulverized lysate was thawed on ice and clarified by centrifugation at 16,000×g for 10 min at 4˚C. Clarified lysate containing 0.5 mg of RNA was digested for 1 h with 750 U of S7 micrococcal nuclease (Roche, 10107921001) at 25˚C. The reaction was quenched by adding EGTA to 6 mM and moved on ice. Undigested lysate containing 0.14 mg of RNA was incubated without nuclease as control. The polysome profile of control samples was obtained by centrifugation through a 7%-47% sucrose gradient at 35,000 rpm for 2.5 hr at 4˚C. The monosome fraction in the digested samples was collected from the sucrose gradient and was purified by hot-phenol extraction. Ribosome-protected mRNA fragments were isolated by size excision on a Novex 15% TBE-Urea gel (Invitrogen, EC6885BOX). Fragments with size ranging from 25 to 35 nucleotides were excised from the gel and extracted. Library generation was performed using the previously described strategy (5) detailed below.

For mRNA-seq performed in parallel with ribosome profiling, total RNA was phenol extracted from the same lysate that was used for ribosome footprinting. Short RNA molecules (sRNA and tRNA) and ribosomal RNA were removed from the total RNA with MEGAclear transcription clean-up kit (Invitrogen, AM1908) and MICROBExpress bacterial mRNA enrichment kit (Ambion, AM1905), respectively. mRNA was randomly fragmented using RNA Fragmentation Reagents (Ambion, AM8740) by incubating at 95˚C for 1 min 45 sec. The fragmented mRNA sample was size selected by running a Novex 15% TBE-Urea gel and excised between 25 and 45 nucleotides. mRNA was extracted and then converted to a complementary DNA library with the same strategy as for ribosome footprints.

Ribo-seq and RNA-seq library generation

Ribosome footprints or RNA fragments were dephosphorylated using 20 units of T4 polynucleotide kinase (New England Biolabs, M0201S) at 37˚C for 1 hr. 20 pmol of RNA were ligated to 1 µL of 100 µM of the oligonucleotide Linker-1 (Supplementary Table S4) 5' rApp/CTGTAGGCACCATCAAT/3'ddC/ using truncated T4 RNA ligase 2 K277Q (New England Biolabs, M0351S) at 37˚C for 3 hr. The ligated products were resolved by gel electrophoresis in a Novex 10% TBE-Urea polyacrylamide gel (Invitrogen, EC6875BOX) and the target ligation products were eluted from the gel. cDNA was generated by reverse transcription using Superscript III (Invitrogen, 18080093) at 50˚C for 30 min and the primer RT, and isolated by size excision on a 10% TBE-Urea polyacrylamide gel. Single-stranded cDNA was circularized with CircLigase ssDNA Ligase (Epicenter, CL4111K) at 60˚C for 2 hr. For ribosome profiling, circularized ssDNA molecules containing ribosomal RNA fragments were further removed using biotin-linked DNA oligos Biot-1 to Biot-4 and MyOne Streptavidin C1 Dynabeads (Invitrogen, 65001). The remaining circularized ssDNA was used as template for PCR amplification using Phusion High-Fidelity DNA polymerase (NEB, M0530S) with the universal primer Uniseq and indexing primers Index (with Illumina 6-base indices 1, 3, 6, 10, 12 and 14). After 8–10 rounds of PCR amplification, the library was size selected in a Novex 8% TBE gel (Invitrogen, EC62152BOX) and the target (168-178 bp for Ribo-seq and 168-188 bp for RNA-seq) products were eluted. The concentration and quality of the library was assessed using an Agilent High Sensitivity DNA Kit (Agilent, 5067-4626). Sequencing was performed on an Illumina HiSeq 4000 system.

Bioinformatic analyses

Calculation of translation efficiency (TE)

To calculate mRNA abundance, the number of mRNA-seq reads mapped to an ORF was normalized by the length of the ORF and the total number of reads that mapped to the genome to yield the number of Reads corresponding to Per Kilobase of message per Million total sequencing reads (RPKM). The protein synthesis rate of individual ORFs was measured by the average ribosome footprint density (Ribo-seq signal) across the ORF body (excluding the first and last 6 amino acids to remove effect of translation initiation and termination), normalized by the total number of Ribo-seq reads that mapped to all ORF bodies across genome. TE of a gene was

calculated by normalizing the protein synthesis rate by its mRNA abundance defined above. Note that when comparing TE of ORFs between different sample (e.g. untreated vs KSG-treated cells), TE values were adjusted by the different overall translation level quantified by the rate of [^{35}S]-L methionine incorporation. Only well-expressed genes were considered for analysis, defined as those with >128 Ribo-seq reads mapped to the ORF for at least one of the samples (untreated and KSG-treated) and with mean RNA-seq signal > 0.2.

Meta-gene analysis of ribosome occupancy

For different samples, the median of ribosome density at each position relative to the first base of ORF start codon was calculated across well-expressed genes and normalized to the mean ribosome density across the ORF body (excluding the first and last 6 amino acids). Well-expressed genes here were defined as those with ORF length > 300 nt, mean RNA-seq signal > 0.2 and mean Ribo-seq signal across ORF body > 0.9, 0.52 and 0.2 for samples untreated, treated with 1 mg/mL and 10 mg/mL KSG, respectively.

Analysis of effect of translation coupling on KSG action

Polycistronic operons are defined from the mRNA-seq data of untreated cells, based on the following criteria: (1) mRNA-seq signals within the region from the last 100 nt of the adjacent upstream gene to the first 100 nt of the downstream gene (including the intergenic region) have a Gini index < 0.35 (indicating signal consistency) and a mean value within 1.5-fold change of the downstream mean mRNA-seq signal (indicating the upstream and downstream genes having similar mRNA levels); (2) distance between the adjacent genes < 200 nt. Overlapping polycistronic genes and non-overlapping polycistronic genes are defined as those with distances from their first bases of ORFs to the last bases of upstream genes <= 0 nt or > 0 nt, respectively.

Identification of leaderless genes and calculation of 5'UTR lengths

Positions of 5' ends of mRNAs were identified from published Rend-seq data (6) based on the following criteria: (1) the location is within 200 nt upstream of expressed ORFs; (2) the 5' end Rend-seq signal of that location is the highest within the 200 nt region and higher than 50-fold of the maximum signal within the ORF. 5'UTR length was calculated from the distance between the 5' end of mRNA and the first base of ORF. Leaderless genes were defined as those actively expressed and with 5'UTR length <= 5 nt.

Generating templates for toeprinting

The DNA templates for the toeprinting analysis of translation of the *hha* and *cspE* genes were generated by a four-primer PCR reaction. The wt *hha* template was generated by PCR-amplifying a portion of the *hha* gene from the genomic DNA of *E. coli*, strain BW25113, using primers hha-FU and hha-R in the presence of the universal primers T7 and NV1 (Table S4). The mutant versions of the *hha* templates with alteration of the nucleotide preceding the start codon were generated by the same approach but the primer hha-FU was replaced with the primers hha-FA, hha-FG and hha-FC for replacing the wt U(-1) with A, G or C, respectively. The sequences of all the constructs used in toeprinting experiments are shown in Table S3. The *cspE* templates were generated using the same strategy except that the hha-specific primers were replaced with the *cspE*-specific primers (cspE-FG, cspE-FG, cspE-FG, cspE-FG, and cspE-R).

The templates for testing KSG-induced translation arrest at the start codons of the genes were generated by a four-primer PCR reaction that combined a gene-specific primer (Table S4) with the universal reverse primer ErmBLR and two additional primers, NV1 and T7. No genomic DNA was used in the reactions. The resulting constructs are shown in Table S3.

Supplementary Figures

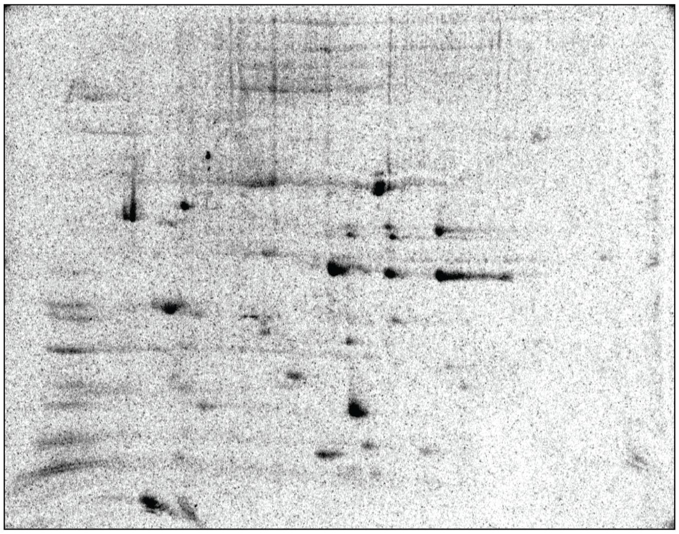

Figure S1. Synthesis of a subset of cellular proteins continues in KSG-treated cells.

The image of the gel shown in Fig. 1E but with the adjusted brightness and contrast to reveal additional spots of radiolabeled proteins.

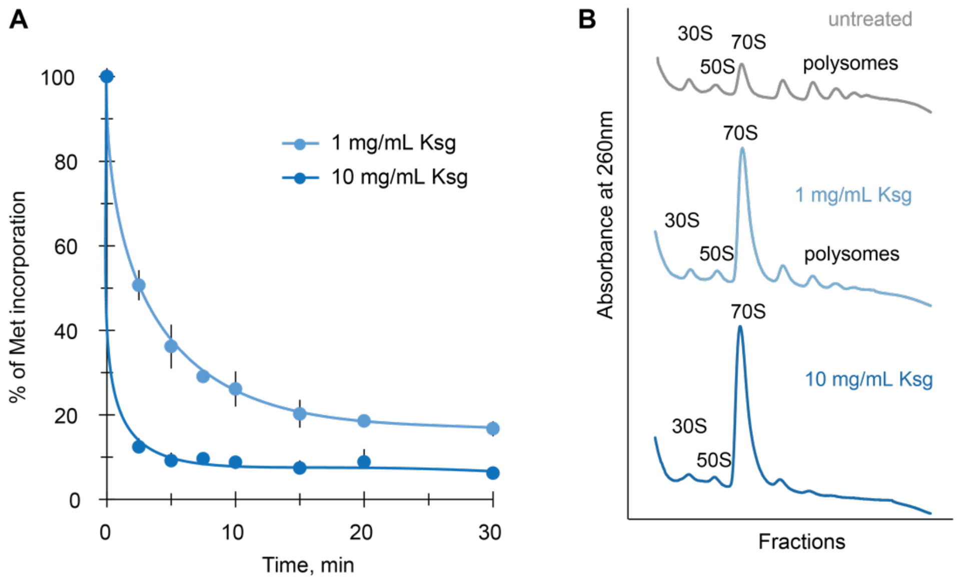

Figure S2. KSG treatment results in moderate disassembly of polysomes

A, Residual translation in *E. coli* MG1655 Δ*gcvB* treated for various times with 1 mg/ml (100x MIC) or 10 mg/ml (1000x MIC) of KSG. KSG MIC for the MG1655 Δ*gcvB* strain is 10 μg/ml. Residual translation was followed by incorporation of [^{35}S]-L-methionine into TCA-precipitable proteins and was normalized to that in the untreated control. **B**, Sucrose gradient centrifugation analysis of the polysomes prepared from MG1655 Δ*gcvB* cells exposed for 2.5 min to 1 mg/mL or 10 mg/mL KSG at 37˚C. Peaks representing 30S and 50S subunits, 70S ribosomes and polysomes are indicated.

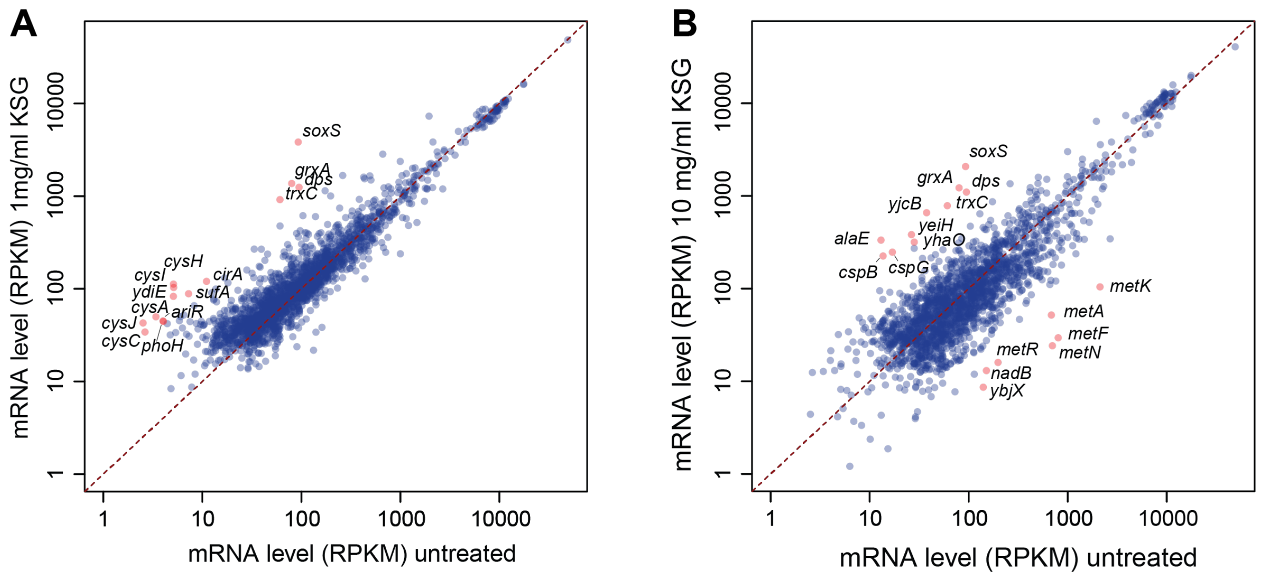

Figure S3. Change of mRNA abundance upon KSG treatment

A, B, Comparison of mRNA level (RPKM from mRNA-seq) in untreated cells to that in cells treated with 1 mg/mL (**A**) or 10 mg/mL (**B**) KSG for 2.5 min. Genes with > 10-fold changes in their mRNA levels upon KSG treatment are highlighted in red and annotated with gene names. Red dash line: y = x diagonal line.

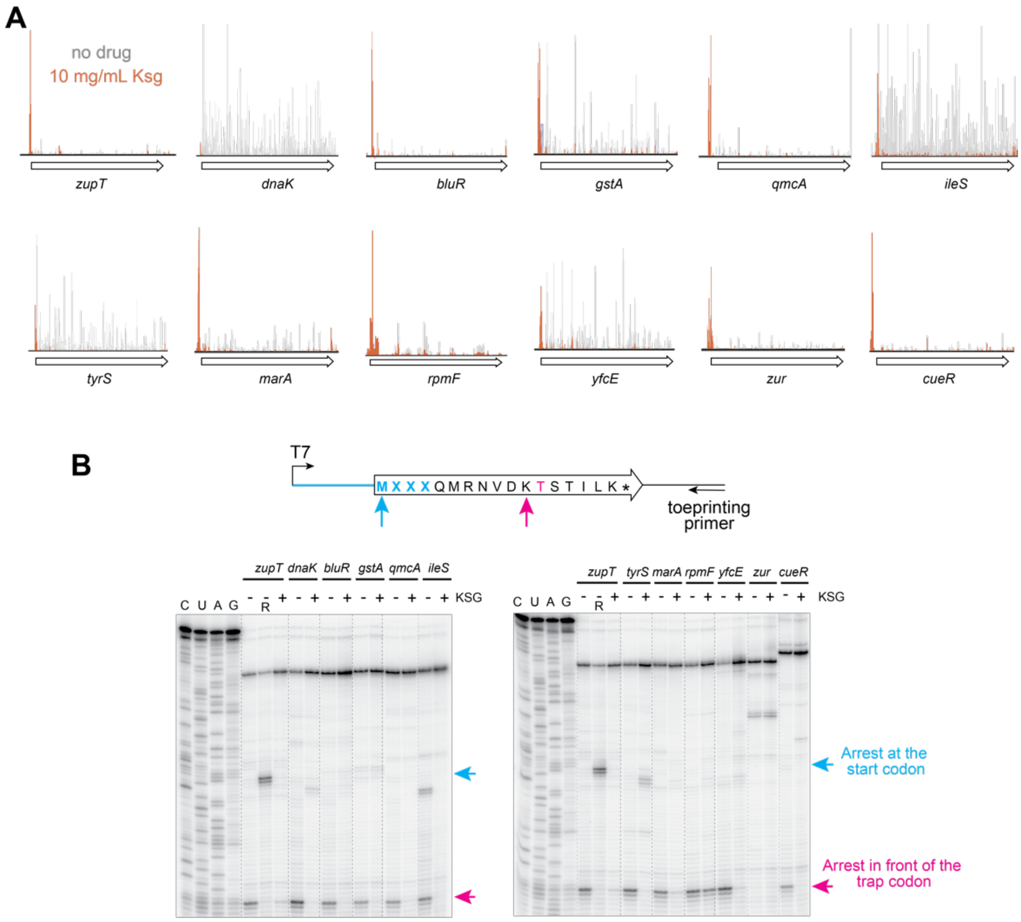

Figure S4. *In vitro* assay does not reproduce KSG-induced ribosome stalling at start codons observed in the living cell.

A, Treatment of *E. coli* with KSG increases ribosome occupancy of start codons of a number of genes. Ribosome density for the untreated control is shown in grey and for the cells treated with 10 mg/mL of KSG – in orange. **B,** *In vitro* toeprinting analysis performed on the same genes in the presence of KSG (1 mM) does not reveal KSG-induced ribosome stalling at start codons. The cartoon illustrates the general scheme of the constructs used in toeprinting. T7 denotes the T7 RNA polymerase promoter. Each template had the first four codons of the tested gene (blue) preceded by 20 nt-long native 5' UTR. The following sequence, containing the trap threonine codon (magenta), was universally present in all the template. Depletion of the reactions of Thr-tRNA was achieved by adding to all the reactions borrelidin, the Thr-RS inhibitor. Retapamulin, known to arrest the ribosome at the start codon, was used as a control antibiotic (lanes marked 'R'). Both borrelidin and retapamulin were present in the reactions at 50 µM.

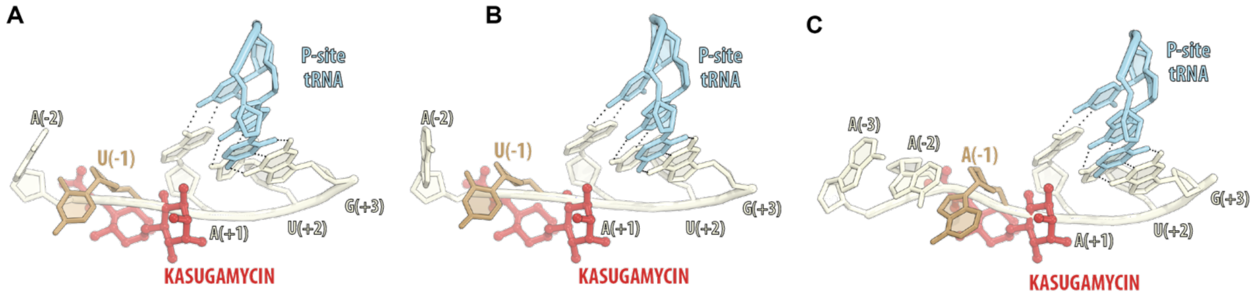

Figure S5. The placement of KSG in the vacant ribosome would clash with mRNA in initiation or elongation translation complexes.

Alignment of structures of the initiating and elongating *E. coli* ribosomes with the *E. coli* ribosome-KSG complex (PDB 4V4H) (7). **A**, 30S IC (PDB 6O7K) (8); **B**, 70S IC (PDB 6O9K) (8); **C**, Elongating ribosome, no E-site tRNA (7K00) (9).

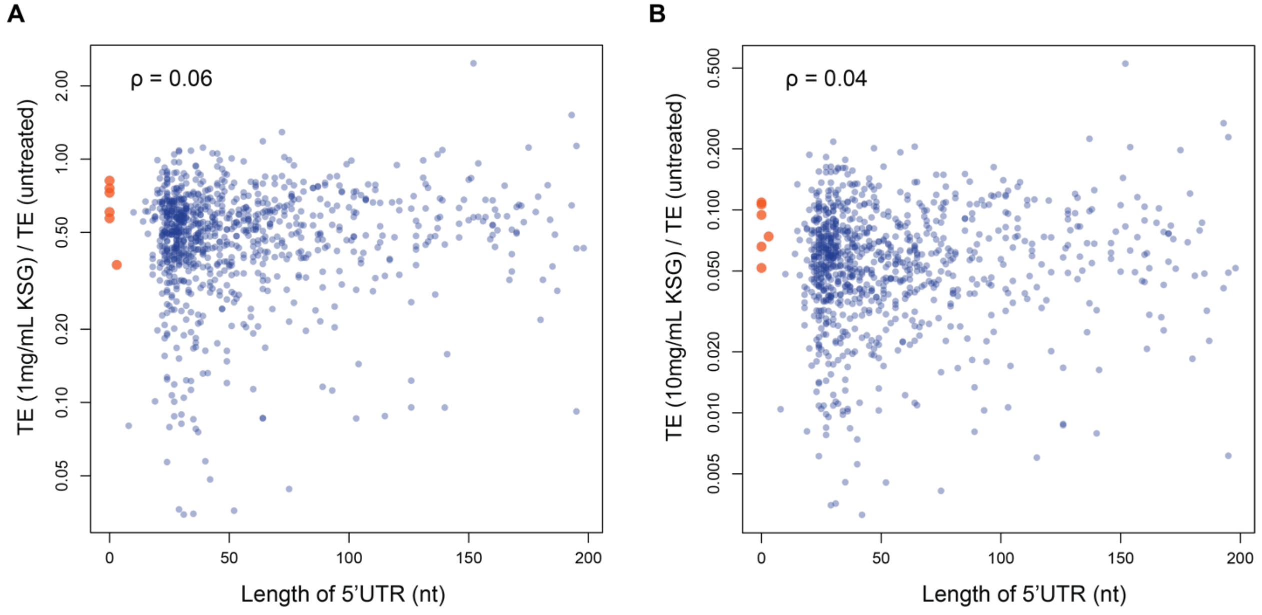

Figure S6. The lack of correlation of the extent of translation inhibition by KSG and the 5' UTR length.

Scatter plot comparisons of the gene TE change in cells exposed to (**A**) 1 mg/mL KSG or (**B**) 10 mg/mL of KSG vs the length of the 5' UTR. Leaderless genes are highlighted in orange. ρ is Spearman's correlation coefficient.

Supplementary Tables

Table S1. Genes with a more than five-fold change in transcription level in kasugamycin treated sample (100x MIC) compared to no drug sample (from RNA-seq data).

Gene name	No drug, RPKM	Ksg 100x MIC, RPKM	Gene function
**Up-regulated in Ksg-treated cells**			
soxS	83	3449	DNA-binding transcriptional dual regulator, participates in the removal of superoxide and nitric oxide and protection from organic solvents and antibiotics
cysH	5	104	phosphoadenosine phosphosulfate reductase
cysI	4	93	sulfite reductase, hemoprotein subunit
grxA	74	1283	reduced glutaredoxin 1
cysJ	2	38	sulfite reductase, flavoprotein subunit complex
trxC	54	826	reduced thioredoxin 2
cysA	3	44	sulfate/thiosulfate ABC transporter ATP binding subunit
dps	84	1129	stationary phase nucleoid component that sequesters iron and protects DNA from damage
sufA	6	81	iron-sulfur cluster insertion protein
cirA	9	109	ferric dihyroxybenzoylserine outer membrane transporter
phoH	4	40	ATP-binding protein
cysN	4	38	sulfate adenylyltransferase subunit 1
fepD	6	48	ferric enterobactin ABC transporter membrane subunit
fpr	30	242	flavodoxin/ferredoxin-NADP+ reductase
entC	7	59	isochorismate synthase
lysA	55	434	diaminopimelate decarboxylase
efeU	15	120	ferrous iron permease
entS	5	41	enterobactin exporter
fumC	11	83	fumarase C
nrdI	13	90	dimanganese-tyrosyl radical cofactor maintenance flavodoxin
fhuA	35	242	ferrichrome outer membrane transporter/phage receptor
nrdH	16	107	glutaredoxin-like protein
iscR	158	1045	DNA-binding transcriptional dual regulator
ahpF	235	1510	alkyl hydroperoxide reductase
fepC	5	30	ferric enterobactin ABC transporter ATP binding subunit
fepG	5	31	ferric enterobactin ABC transporter membrane subunit
fes	6	34	ferric enterobactin esterase
katG	93	557	catalase/hydroperoxidase
ygaW	12	69	L-alanine exporter
sufB	7	39	Fe-S cluster scaffold complex subunit
yhaO	26	145	putative D/L-serine transporter
puuD	8	39	γ-glutamyl-γ-aminobutyrate hydrolase
metR	177	904	DNA-binding transcriptional dual regulator
**Down-regulated in Ksg-treated cells**			
asnA	120	20	asparagine synthetase A

Table S2. Genes with a more than five-fold change in transcription level in kasugamycin treated sample (1000x MIC) compared to no drug sample (from RNA-seq data).

Gene name	No drug, RPKM	Ksg 1000x MIC, RPKM	Gene function
**Up-regulated in Ksg-treated cells**			
ygaW	12	302	L-alanine exporter
soxS	83	1885	DNA-binding transcriptional dual regulator, participates in the removal of superoxide and nitric oxide and protection from organic solvents and antibiotics
yjcB	34	607	uncharacterized protein
cspB	13	224	Qin prophage; cold shock-like protein
grxA	74	1153	reduced glutaredoxin 1
cspG	16	241	cold shock protein
yeiH	24	348	inner membrane protein
trxC	54	717	reduced thioredoxin 2
dps	84	1009	stationary phase nucleoid component that sequesters iron and protects DNA from damage
yhaO	26	291	putative D/L-serine transporter
yhdL	26	242	alternative ribosome-rescue factor A
intB	30	195	KpLE2 phage-like element; putative integrase
yffL	5	30	CPZ-55 prophage; uncharacterized protein
zntR	14	86	DNA-binding transcriptional activator
yhiP	5	32	dipeptide/tripeptide:H+ symporter
phoH	4	20	ATP-binding protein
yafT	13	72	lipoprotein
ylaC	40	212	putative inner membrane protein
ahpF	235	1232	alkyl hydroperoxide reductase
yfhL	38	196	lipoprotein
iscR	158	788	DNA-binding transcriptional dual regulator
**Down-regulated in Ksg-treated cells**			
yeeF	1096	219	putrescine:H+ symporter
tpiA	388	77	triose-phosphate isomerase
fes	6	1	ferric enterobactin esterase
cpxP	50	10	periplasmic protein, involved in resistance to extracytoplasmic stresses
metL	345	67	fused aspartate kinase/homoserine dehydrogenase 2
aroG	154	29	3-deoxy-7-phosphoheptulonate synthase
pyrC	101	19	dihydroorotase
guaB	607	113	inosine 5'-monophosphate dehydrogenase
ybdH	136	25	hydroxycarboxylate dehydrogenase A
nmpC	345	63	DLP12 prophage; putative outer membrane porin
metC	104	19	cystathionine β-lyase / L-cysteine desulfhydrase / alanine racemase
dsdA	42	8	D-serine ammonia-lyase
nupC	119	21	nucleoside:H+ symporter
asnA	120	20	asparagine synthetase A
mmuM	127	21	CP4-6 prophage; homocysteine S-methyltransferase
fbaA	590	98	fructose-bisphosphate aldolase class II
solA	37	6	N-methyl-L-tryptophan oxidase
metI	688	112	L-methionine/D-methionine ABC transporter membrane subunit
yeaD	27	4	D-glycero-β-D-manno-heptose-1,7-bisphosphate 7-phosphatase
metB	290	42	O-succinylhomoserine(thiol)-lyase / O-succinylhomoserine lyase
rstB	20	3	sensory histidine kinase

eptA	26	4	phosphoethanolamine transferase EptA
rstA	26	4	DNA-binding transcriptional regulator
gapA	2415	312	glyceraldehyde-3-phosphate dehydrogenase
fiu	14	2	iron catecholate outer membrane transporter
mmuP	102	11	CP4-6 prophage; S-methyl-L-methionine transporter
ybdL	66	7	methionine-oxo-acid transaminase, PLP-dependent
nadB	136	12	L-aspartate oxidase
metR	177	15	DNA-binding transcriptional dual regulator
metA	625	48	homoserine O-succinyltransferase
ybjX	125	8	putative protein
metK	1898	94	methionine adenosyltransferase
metF	730	26	5,10-methylenetetrahydrofolate reductase
metN	624	22	L-methionine/D-methionine ABC transporter ATP binding subunit

Table S3. The nucleotide sequences of the DNA constructs used in the toeprinting experiments

**gene**	**Sequence** [a)]
hha(U)	TACATTAATACGACTCACTATAGGGTATTACAACCATAGGTAGAAG**T**ATGTCCGAAAAAC<br>CTTTAACGAAAACCGATTATTTAATGCGTTTACGTCGTTGCCTAAGTGATAGAATTCTATC<br>CCTGCTTAGAGCCCTCTCTCCCTAGCCTCCGCTCGTTAATAAGCAAAATTCATTATAACC
hha(A)	TACATTAATACGACTCACTATAGGGTATTACAACCATAGGTAGAAG**A**ATGTCCGAAAAAC<br>CTTTAACGAAAACCGATTATTTAATGCGTTTACGTCGTTGCCTAAGTGATAGAATTCTATC<br>CCTGCTTAGAGCCCTCTCTCCCTAGCCTCCGCTCGTTAATAAGCAAAATTCATTATAACC
hha(G)	TACATTAATACGACTCACTATAGGGTATTACAACCATAGGTAGAAG**G**ATGTCCGAAAAA<br>CCTTTAACGAAAACCGATTATTTAATGCGTTTACGTCGTTGCCTAAGTGATAGAATTCTAT<br>CCCTGCTTAGAGCCCTCTCTCCCTAGCCTCCGCTCGTTAATAAGCAAAATTCATTATAACC
hha(C)	TACATTAATACGACTCACTATAGGGTATTACAACCATAGGTAGAAG**C**ATGTCCGAAAAAC<br>CTTTAACGAAAACCGATTATTTAATGCGTTTACGTCGTTGCCTAAGTGATAGAATTCTATC<br>CCTGCTTAGAGCCCTCTCTCCCTAGCCTCCGCTCGTTAATAAGCAAAATTCATTATAACC
cspE(G)	TACATTAATACGACTCACTATAGGGATTGGTGTACTGGTAACCGACACAGCATTTGTGTC<br>TATTTTTCATGTAAAGGTAATTTT**G**ATGTCTAAGATTAAAGGTAACGTTAAGTGGTTTAAT<br>GAGTCCAAAGGATTCGGTCTAAGTGATAGAATTCTATCCCTGCTTAGAGCCCTCTCTCCCT<br>AGCCTCCGCTCGTTAATAAGCAAAATTCATTATAACC
cspE (A)	TACATTAATACGACTCACTATAGGGATTGGTGTACTGGTAACCGACACAGCATTTGTGTC<br>TATTTTTCATGTAAAGGTAATTTT**A**ATGTCTAAGATTAAAGGTAACGTTAAGTGGTTTAAT<br>GAGTCCAAAGGATTCGGTCTAAGTGATAGAATTCTATCCCTGCTTAGAGCCCTCTCTCCCT<br>AGCCTCCGCTCGTTAATAAGCAAAATTCATTATAACC
cspE (U)	TACATTAATACGACTCACTATAGGGATTGGTGTACTGGTAACCGACACAGCATTTGTGTC<br>TATTTTTCATGTAAAGGTAATTTT**T**ATGTCTAAGATTAAAGGTAACGTTAAGTGGTTTAAT<br>GAGTCCAAAGGATTCGGTCTAAGTGATAGAATTCTATCCCTGCTTAGAGCCCTCTCTCCCT<br>AGCCTCCGCTCGTTAATAAGCAAAATTCATTATAACC
cspE (C)	TACATTAATACGACTCACTATAGGGATTGGTGTACTGGTAACCGACACAGCATTTGTGTC<br>TATTTTTCATGTAAAGGTAATTTT**C**ATGTCTAAGATTAAAGGTAACGTTAAGTGGTTTAAT<br>GAGTCCAAAGGATTCGGTCTAAGTGATAGAATTCTATCCCTGCTTAGAGCCCTCTCTCCCT<br>AGCCTCCGCTCGTTAATAAGCAAAATTCATTATAACC
zupT	TACATTAATACGACTCACTATAGGGAGATTTATAAGGAGTTGTCGATGTCAGTACCTCAA<br>ATGCGTAATGTAGATAAAACATCTACTATTTTGAAATAAGTGATAGAATTCTATCGTTAA<br>TAAGCAAAATTCATTATAACC
dnaK	TACATTAATACGACTCACTATAGGGTATATAGTGGAGACGTTTAGATGGGTAAAATACAA<br>ATGCGTAATGTAGATAAAACATCTACTATTTTGAAATAAGTGATAGAATTCTATCGTTAA<br>TAAGCAAAATTCATTATAACC
bluR	TACATTAATACGACTCACTATAGGGTGTGACCGCAAGGAGGAATTGTGGCTTATTACCAA<br>ATGCGTAATGTAGATAAAACATCTACTATTTTGAAATAAGTGATAGAATTCTATCGTTAA<br>TAAGCAAAATTCATTATAACC
gstA	TACATTAATACGACTCACTATAGGGACAGACAAAAGGAGTTACCGATGAAATTGTTCCAA<br>ATGCGTAATGTAGATAAAACATCTACTATTTTGAAATAAGTGATAGAATTCTATCGTTAA<br>TAAGCAAAATTCATTATAACC
qmcA	TACATTAATACGACTCACTATAGGGTACAACAGGAGGTTTTTCCGATGCTTATCTTTCAAA<br>TGCGTAATGTAGATAAAACATCTACTATTTTGAAATAAGTGATAGAATTCTATCGTTAAT<br>AAGCAAAATTCATTATAACC
ileS	TACATTAATACGACTCACTATAGGGAATACGGAACCGAGAATCTGATGAGTGACTATCAA<br>ATGCGTAATGTAGATAAAACATCTACTATTTTGAAATAAGTGATAGAATTCTATCGTTAA<br>TAAGCAAAATTCATTATAACC

tyrS	TACATTAATACGACTCACTATAGGGTTATATACATGGAGATTTTGATGGCAAGCAGTCAA ATGCGTAATGTAGATAAAACATCTACTATTTTGAAATAAGTGATAGAATTCTATCGTTAA TAAGCAAAATTCATTATAACC
marA	TACATTAATACGACTCACTATAGGGAACAAAAAAGAGGTATGACGATGTCCAGACGCCA AATGCGTAATGTAGATAAAACATCTACTATTTTGAAATAAGTGATAGAATTCTATCGTTA ATAAGCAAAATTCATTATAACC
rpmF	TACATTAATACGACTCACTATAGGGGTAATTGAGGAGTAAGGTCCATGGCCGTACAACAA ATGCGTAATGTAGATAAAACATCTACTATTTTGAAATAAGTGATAGAATTCTATCGTTAA TAAGCAAAATTCATTATAACC
yfcE	TACATTAATACGACTCACTATAGGGGTTTTTTCGACGAGGAGTGAGTGATGAAACTGCAA ATGCGTAATGTAGATAAAACATCTACTATTTTGAAATAAGTGATAGAATTCTATCGTTAA TAAGCAAAATTCATTATAACC
zur	TACATTAATACGACTCACTATAGGGCCCCCACTTTGAGGTGCCCGATGGAAAAGACCCAA ATGCGTAATGTAGATAAAACATCTACTATTTTGAAATAAGTGATAGAATTCTATCGTTAA TAAGCAAAATTCATTATAACC
cueR	TACATTAATACGACTCACTATAGGGAGCACAGGAGGCGTTGCGCGAACGATGAACATCA GCCAAATGCGTAATGTAGATAAAACATCTACTATTTTGAAATAAGTGATAGAATTCTATC GTTAATAAGCAAAATTCATTATAACC

[a] T7 promoter is orange, 5’ UTR and the 5’ coding region of the gene is purple (with the coding region underlined) and the universal sequences added by PCR are black. In each template, the start codon and the in-frame stop codon are shown in red. The sequence complementary to the universal toeprinting primer NV1 is blue.

Table S4. Oligonucleotides used in the study

Oligo	Sequence (5' –> 3')
Linker-1	rApp/CTGTAGGCACCATCAAT/ddC
RT	/5Phos/AGATCGGAAGAGCGTCGTGTAGGGAAAGAGTGT/iSp18/CAAGCAGAAGACGGCATACGAGATATTGATGGTGCCTACAG [a)]
Biot-1	/5Biosg/TCATCTCCGGGGGTAGAGCACTGTTTCG [b)]
Biot-2	/5Biosg/GGCTAAACCATGCACCGAAGCTGCGGCAG
Biot-3	/5Biosg/AAGGCTGAGGCGTGATGACGAGGCACT
Biot-4	/5Biosg/CGGTGCTGAAGCAACAAATGCCCTGCTT
Uniseq	CAAGCAGAAGACGGCATACGA
Index 1-atcacg	AATGATACGGCGACCACCGAGATCTACACGATCGGAAGAGCACACGTCTGAACTCCAGTCAC**atcacg**ACACTCTTTCCCTACAC [c)]
Index 3-ttaggc	AATGATACGGCGACCACCGAGATCTACACGATCGGAAGAGCACACGTCTGAACTCCAGTCAC**ttaggc**ACACTCTTTCCCTACAC [c)]
Index 6-gccaat	AATGATACGGCGACCACCGAGATCTACACGATCGGAAGAGCACACGTCTGAACTCCAGTCAC**gccaat**ACACTCTTTCCCTACAC [c)]
Index 10-tagctt	AATGATACGGCGACCACCGAGATCTACACGATCGGAAGAGCACACGTCTGAACTCCAGTCAC**tagctt**ACACTCTTTCCCTACAC [c)]
Index 12-cttgta	AATGATACGGCGACCACCGAGATCTACACGATCGGAAGAGCACACGTCTGAACTCCAGTCAC**cttgta**ACACTCTTTCCCTACAC [c)]
Index 14-agttcc	AATGATACGGCGACCACCGAGATCTACACGATCGGAAGAGCACACGTCTGAACTCCAGTCAC**agttcc**ACACTCTTTCCCTACAC [c)]
hha-FU	TACATTAATACGACTCACTATAGGGTATTACAACCATAGGTAGAAG**T**ATGTCCGA [d)]
hha-FA	TACATTAATACGACTCACTATAGGGTATTACAACCATAGGTAGAAG**A**ATGTCCGA [d)]
hha-FG	TACATTAATACGACTCACTATAGGGTATTACAACCATAGGTAGAAG**G**ATGTCCGA [d)]
hha-FC	TACATTAATACGACTCACTATAGGGTATTACAACCATAGGTAGAAG**C**ATGTCCGA [d)]
hha-R	AGAGAGGGCTCTAAGCAGGGATAGAATTCTATCACTTAGGCAACGACGTAAACGCATTAAATAATCGGTTV
cspE-FG	TACATTAATACGACTCACTATAGGGTTTCATGTAAAGGTAATTTT**G**ATGTCTAA [d)]
cspE-FA	TACATTAATACGACTCACTATAGGGTTTCATGTAAAGGTAATTTT**A**ATGTCTAA [d)]
cspE-FU	TACATTAATACGACTCACTATAGGGTTTCATGTAAAGGTAATTTT**T**ATGTCTAA [d)]
cspE-FC	TACATTAATACGACTCACTATAGGGTTTCATGTAAAGGTAATTTT**C**ATGTCTAA [d)]
cspE-R	AGAGAGGGCTCTAAGCAGGGATAGAATTCTATCACTTAGACCGAATCCTTTGGACTCATTAAACCACT
zupT-F	TACATTAATACGACTCACTATAGGGAGATTTATAAGGAGTTGTCGATGTCAGTACCTCAAATGCGTAATGTAGATAAAACATCTAC
dnaK-F	TACATTAATACGACTCACTATAGGGTATATAGTGGAGACGTTTAGATGGGTAAAATACAAATGCGTAATGTAGATAAAACATCTAC
bluR-F	TACATTAATACGACTCACTATAGGGTGTGACCGCAAGGAGGAATTGTGGCTTATTACCAAATGCGTAATGTAGATAAAACATCTAC
gstA-F	TACATTAATACGACTCACTATAGGGACAGACAAAAGGAGTTACCGATGAAATTGTTCCAAATGCGTAATGTAGATAAAACATCTAC
qmcA-F	TACATTAATACGACTCACTATAGGGTACAACAGGAGGTTTTTCCGATGCTTATCTTTCAAATGCGTAATGTAGATAAAACATCTAC
ileS-F	TACATTAATACGACTCACTATAGGGAATACGGAACCGAGAATCTGATGAGTGACTATCAAATGCGTAATGTAGATAAAACATCTAC
tyrS-F	TACATTAATACGACTCACTATAGGGTTATATACATGGAGATTTTGATGGCAAGCAGTCAAATGCGTAATGTAGATAAAACATCTAC
marA-F	TACATTAATACGACTCACTATAGGGAACAAAAAAGAGGTATGACGATGTCCAGACGCCAAATGCGTAATGTAGATAAAACATCTAC
rpmF-F	TACATTAATACGACTCACTATAGGGGTAATTGAGGAGTAAGGTCCATGGCCGTACAACAAATGCGTAATGTAGATAAAACATCTAC
yfcE-F	TACATTAATACGACTCACTATAGGGGTTTTTCGACGAGGAGTGAGTGATGAAACTGCAAATGCGTAATGTAGATAAAACATCTAC

zur-F	TACATTAATACGACTCACTATAGGGCCCCCACTTTGAGGTGCCCGATGGAAAAGACCCAAATGCGTAATGTAGATAAAACATCTAC
cueR-F	TACATTAATACGACTCACTATAGGGAGCACAGGAGGCGTTGCGCGAACGATGAACATCAGCCAAATGCGTAATGTAGATAAAACATCTAC
ermBLR	GGTTATAATGAATTTTGCTTATTAACGATAGAATTCTATCACTTATTTCAAAATAGTAGATGTTTTATCTACATTACG
NV1	GGTTATAATGAATTTTGCTTATTAAC
T7	TACATTAATACGACTCACTATAGGG

[a)] iSp18 is an 18-atom hexa-ethyleneglycol spacer (IDT)

[b)] /5Biosg/ is 5' biotin modification (IDT)

[c)] PCR primer for Ribo-seq and RNA-seq library preparation with the Illumina 6-base indices highlighted

[d)] The nucleotide preceding the start codon is bold and start codon is underlined

Supplementary references

1. D.J. Dwyer, P.A. Belenky, J.H. Yang, I.C. MacDonald, J.D. Martell *et al.* Antibiotics induce redox-related physiological alterations as part of their lethality. *Proc Natl Acad Sci USA*, **111**, E2100-2109 (2014)
2. S. Meydan, J. Marks, D. Klepacki, V. Sharma, P.V. Baranov *et al.* Retapamulin-assisted ribosome profiling reveals the alternative bacterial proteome. *Molec Cell*, **74**, 481-493 (2019)
3. M.M. Bradford. A rapid and sensitive method for the quantitation of microgram quantities of protein utilizing the principle of protein-dye binding. *Analyt Biochem*, **72**, 248-254 (1976)
4. H. Schägger, G. von Jagow. Tricine-sodium dodecyl sulfate-polyacrylamide gel electrophoresis for the separation of proteins in the range from 1 to 100 kDa. *Analyt Biochem*, **166**, 368-379 (1987)
5. G.W. Li, D. Burkhardt, C. Gross, J.S. Weissman. Quantifying absolute protein synthesis rates reveals principles underlying allocation of cellular resources. *Cell*, **157**, 624-635 (2014)
6. J.B. Lalanne, J.C. Taggart, M.S. Guo, L. Herzel, A. Schieler *et al.* Evolutionary convergence of pathway-specific enzyme expression stoichiometry. *Cell*, **173**, 749-761 e738 (2018)
7. B.S. Schuwirth, J.M. Day, C.W. Hau, G.R. Janssen, A.E. Dahlberg *et al.* Structural analysis of kasugamycin inhibition of translation. *Nat Struct Molec Biol*, **13**, 879-886 (2006)
8. S. Kaledhonkar, Z. Fu, K. Caban, W. Li, B. Chen *et al.* Late steps in bacterial translation initiation visualized using time-resolved cryo-EM. *Nature*, **570**, 400-404 (2019)
9. Z.L. Watson, F.R. Ward, R. Meheust, O. Ad, A. Schepartz *et al.* Structure of the bacterial ribosome at 2 A resolution. *eLife*, **9** (2020)
